# Synergistic Activity of Natural Antimicrobials Against Common Fish Spoilage and Pathogenic Bacteria

**DOI:** 10.1002/fsn3.72025

**Published:** 2026-07-17

**Authors:** Paul G. Kamau, Malco C. Cruz‐Romero, Paola C. Alzate, Michael A. Morris, Joe P. Kerry

**Affiliations:** ^1^ Food Packaging and Materials Science Group, School of Food & Nutritional Sciences University College Cork Cork Ireland; ^2^ AMBER and the School of Chemistry Trinity College Dublin Dublin 2 Ireland

**Keywords:** chitosan, essential oils, fractional inhibitory concentration index, natural antimicrobials, organic acids, synergistic effect

## Abstract

This study examined the antimicrobial activity (AA) of a suite of natural antimicrobials (NAM): low molecular weight chitosan (CS), essential oils (EOs; thyme, oregano, garlic, sage, laurel, marjoram and rosemary) and commercially available mixtures of weak organic acids (Articoat (ART) and Sulac (SUL)), tested individually and in combinations against common fish spoilage ( *Brochothrix thermosphacta* ( *B. thermosphacta* ), Lactic acid bacteria (LAB) and *Pseudomonas fluorescens* ( *P. fluorescens* )), pathogenic ( *Staphylococcus aureus* ( *S. aureus* ), *Listeria innocua* ( *L. innocua* ) and *Escherichia coli* ( *E. coli* )) bacteria and raw salmon microbiota. Individually, CS showed the strongest AA across all bacteria, with a minimum inhibitory concentration (MIC) of 0.125 mg/mL, outperforming all tested EOs, ART, and SUL. Among the 40 combinations tested (ART–EOs, CS–EOs, SUL–EOs and EO–EO), CS–garlic EO (GEO) had the strongest synergistic effect, significantly (*p* < 0.05) reducing CS and GEO MICs by 16‐ and 8‐fold, respectively, against *S. aureus, L. innocua, B. thermosphacta* and LAB when compared to the individual MICs; a comparable synergistic effect was noticed for SUL combined with GEO or sage EO, significantly lowering (*p* < 0.05) SUL and EO MICs by 17‐ and 8‐fold, respectively against the same bacteria. Oregano (OEO)–thyme (TEO), ART‐OEO and ART‐TEO demonstrated the strongest synergistic effect, reducing required concentrations (*p* < 0.05) by up to 8‐fold, against *E. coli, P. fluorescens* and salmon microbiota compared to their individual MICs. Overall, synergistic NAM combinations enhanced AA while substantially reducing antimicrobial concentrations, highlighting their potential for application in foods and packaging to extend shelf‐life, support clean‐label preservation, reduce waste and enhance sustainability.

## Graphical abstract

This study systematically investigated the antimicrobial activity of a range of natural antimicrobials, applied individually and in combination, against key fish spoilage microorganisms, foodborne pathogens, and native salmon microbiota. The aim was to identify effective synergistic combinations that reduce the concentrations required to inhibit bacterial growth, thereby enhancing their potential for use in food preservation and packaging applications.

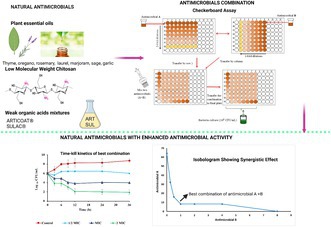

## 1 Introduction

The global food system is increasingly challenged by the dual imperatives of ensuring food safety and minimizing food waste, both of which are critical for public health, environmental sustainability and the achievement of global food security (Cava‐Roda et al. 2021; Quinto et al. 2019; WHO 2024). In parallel, the increasing demand for sustainable, fresh‐like, minimally processed and clean label food products has driven interest in the use of natural antimicrobials (NAM) for food preservation (Kamau et al. 2025; Sullivan et al. 2017). NAM such as EOs, CS and commercially available mixtures of weak organic acids and their salts (ART and SUL) have been used as naturally occurring preservatives in food and beverages. Their capacity to extend the shelf‐life of perishable products, particularly meat and fish, has been well documented and is attributed to their broad‐spectrum antimicrobial activity against both spoilage organisms and foodborne pathogens (Cruz‐Romero et al. 2013; Davidson et al. 2013; Fournomiti et al. 2015; Gurtler and Garner 2022; Hassan et al. 2019; Kamau et al. 2025; Kerry 2012; Nedic et al. 2021; Pereira et al. 2023; Rubio et al. 2018; Sullivan et al. 2017; Sullivan et al. 2020; Teixeira et al. 2013; Tosun 2021; Wang et al. 2019; Xing et al. 2021). However, the relatively high concentrations of individual NAM required to achieve microbial safety and extend shelf‐life limit their practical application in foods, as such levels can compromise product quality by causing sensory defects, including off‐flavors or increased acidity (Cho et al. 2020; Sullivan et al. 2017; Tiwari et al. 2009). Their effectiveness is further constrained by reduced activity at higher pH values and by interactions with food matrices that may bind or neutralize NAM, thereby diminishing their antimicrobial potential (Kovanda et al. 2019; Taylor and Doores 2020).

NAM such as EO, CS, or weak organic acids exhibit different antimicrobial modes of action. Consequently, combining individual NAM can broaden their overall antimicrobial spectrum by generating multi‐mechanistic antimicrobial formulations. Such synergistic interactions have been shown to enhance AA, improve efficacy at lower concentrations, reduce toxicity and sensory impacts in applied foods, and reduce overall application costs (Fratini et al. 2025; Hassoun and Emir Çoban 2017; Hyldgaard et al. 2012; Kamau et al. 2025; Yuan et al. 2016). Several studies have demonstrated that combining EOs can produce synergistic activity against foodborne pathogens and spoilage microorganisms (Abu‐Hussien et al. 2025; Bassolé and Juliani 2012; Fratini et al. 2025; Hu et al. 2021; Kunová et al. 2021; Meerasri et al. 2024; Nedic et al. 2021; dos Santos et al. 2024; Soulaimani 2025; Zhang et al. 2024). For example, synergistic effects against *Pseudomonas aeruginosa, Listeria monocytogenes* ( *L. monocytogenes* ), and *S. aureus* have been reported for combinations of thyme and oregano EOs (Křížek et al. 2018; Meerasri et al. 2024; Nedic et al. 2021). In addition, synergistic activity has been widely observed when CS was combined with EOs such as oregano, lemon, thyme, cinnamon, clove, sage, and rosemary, resulting in enhanced inhibition of foodborne pathogens including *S. aureus* , *E. coli* , and *Salmonella Typhimurium* (Aref et al. 2022; Avila‐Sosa et al. 2025; Chaudhari et al. 2023; Dhanasekaran et al. 2024; Ehsani et al. 2020; Gaba et al. 2022; Granata et al. 2021; Nawaz et al. 2020; Parichanon et al. 2023; Shetta et al. 2024). Synergistic combinations of weak organic acids have also been explored; for instance, acetic acid combined with citric or ascorbic acid has demonstrated enhanced antimicrobial efficacy against fish spoilage bacteria (Anagnostopoulos et al. 2023; Lee et al. 2019). Similarly, a combination of caprylic, sorbic, and caproic acids exhibited synergistic activity against a broad range of *Campylobacter* spp. (Peh et al. 2020). Moreover, synergistic effect between oregano EO and acetic acid has been reported against pathogenic *E. coli* and *L. monocytogenes* (Ijabadeniyi et al. 2020; de Souza et al. 2009).

Although NAM combinations have shown considerable promise, to the best of our knowledge, comprehensive studies that systematically compare multiple NAM classes under uniform experimental conditions are still scarce. Moreover, as demonstrated in our previous study, the preparation method of NAM, particularly EOs, critically influences their AA (Kamau et al. 2026). This lack of comparative data represents a significant gap in identifying the most effective antimicrobial combinations for practical applications in fish preservation, particularly for inhibiting key fish spoilage microorganisms and extending the shelf‐life of these highly perishable products. Therefore, the objective of this study was to evaluate, using an optimized preparation method, the AA of a diverse range of NAM, including EOs (thyme, oregano, garlic, sage, laurel, marjoram, and rosemary), commercially available mixtures of weak organic acids (ART) and SUL, and CS. These NAM were assessed, both individually and in combination, against common fish spoilage and pathogenic bacteria (*S. aureus, L. innocua*, LAB, *E. coli* , and *P. fluorescens* ) and raw salmon microbiota.

## 2 Materials and Methods

### 2.1 Materials

Low molecular weight chitosan (CS) (MW: 50–190 kDa, ≥ 75% deacetylated), aqueous acetic acid, hydrogen peroxide (H_2_O_2_: 30%), ethanol (≥ 99.5%) and Tween 80 were all purchased from Sigma‐Aldrich (St. Louis, Missouri, USA). Garlic, sage, marjoram, thyme, laurel, rosemary and oregano EOs were obtained from Lionel Hitchen Ltd. (Barton Stacey, Hampshire, UK). The chemical compositions of the EOs used in this study were obtained from manufacturer‐provided product specification sheets. These specifications are based on gas chromatographic analyses performed by the suppliers and report relative percentage ranges of the major constituents and are presented in Table S1. Commercially available mixtures of weak organic acids used as meat coatings; ART (sodium diacetate, lactic acid, acetic acid, citric acid, in pectin and water) and SUL (lactic acid and calcium sulphate) were obtained from Chemital (Barcelona, Spain). Media for microbiological analysis, including Plate Count Agar (PCA), Maximum Recovery Diluent (MRD), Mueller‐Hinton Broth (MHB), Mueller‐Hinton Agar (MHA), De Man, Rogosa and Sharpe (MRS) agar, Streptomycin Thallous Acetate Actidione (STAA) agar base with selective supplement SR0151E (Streptomycin Sulphate and Thallous Acetate) and oxidase touch Sticks, were all purchased from Oxoid (Basingstoke, Hampshire, England). Stomacher bags (with filters) were obtained from Interscience (France). Raw vacuum skin‐packed salmon fillets were sourced locally. Sterile distilled water was used in all experiments.

### 2.2 Preparation of Antimicrobial Solutions

#### 2.2.1 EO Emulsions

Garlic, sage, marjoram, thyme, laurel, rosemary, or oregano EO emulsions were prepared using the ultrasonication method as previously described by Kamau et al. (2026). Briefly, EOs (0.5%, v/v) were dissolved in 50% (v/v) ethanol to which Tween 80 (non‐ionic) surfactant was added at an oil‐to‐surfactant ratio of 10:1 (v/v), and solutions were sonicated using an EpiShear probe sonicator (Active Motif, UK) fitted with a 2 mm probe, operated at 20 kHz and 80% amplitude. Sonication totalled 60 s, delivered in two 30 s intervals separated by 10 s pauses. To prevent the temperature increase of the sonicated samples, tubes were placed on ice during sonication.

#### 2.2.2 CS Solution

Solution of 0.1% CS was prepared as previously outlined by Sullivan et al. (2020). Briefly, 0.1% CS low molecular weight CS was dissolved in a 1% v/v aqueous acetic acid solution and stirred on a magnetic stirrer at 25°C until fully dissolved.

#### 2.2.3 Commercially Available Mixtures of Weak Organic Acids

The commercial mixtures of organic acids; ART and SUL, were diluted with sterile distilled water at concentrations of 20% and 3%, respectively, as recommended by the supplier.

### 2.3 Particle Size and Polydispersity Index (PDI) of EO Emulsions

The average particle size and PDI of EO emulsions were determined using a Dynamic Light Scattering (DLS) analyzer (Malvern, UK). To minimize multiple scattering effects, prior to measurement, samples were tenfold diluted with 50% (v/v) ethanol. The diluted emulsions were loaded into disposable cuvettes (ZEN0040) and analyzed for 15 runs at 25°C using a backscattering angle of 173° and a 1.5 refractive index. Each EO emulsion was analyzed in duplicate in three independent experiments. Particle size distributions were calculated using the Mark–Houwink and Smoluchowski models.

### 2.4 Bacterial Strains

#### 2.4.1 Pure Strains

In this study, pure strains of *E. coli* (NCIMB 11943), *S. aureus* (NCDO 949), *L. innocua* (FPL 012), and *P. fluorescens* (NCIMB 9046) were used. All strains were maintained on PCA slants at 4°C until use. To obtain cultures in the logarithmic growth phase, a loopful of each strain was inoculated into sterile tubes (Sterilin, UK) containing 10 mL of sterile doublestrength MHB and incubated on an orbital shaker (Innova 2300, New Brunswick, Germany) at 170 rpm for 18 h at 37°C ( *E. coli* , *S. aureus* , and *L. innocua* ) or 30°C ( *P. fluorescens* ). This was followed by a secondary incubation step, in which 0.2 mL of the overnight culture was transferred into fresh 10 mL sterile MHB and incubated for an additional 1 h at 37°C ( *E. coli* , *S. aureus* , and *L. innocua* ) or 30°C ( *P. fluorescens* ) to ensure active exponential‐phase growth.

#### 2.4.2 Raw Salmon Microbiota and Isolated Strains of LAB and *B. thermosphacta*

For the isolation of microbiota of raw salmon, two skin‐on salmon darnes were aseptically transferred into a sterile plastic jar and blended for 30 s using a Waring Blender (Christison, UK). Subsequently, 25 g of the resulting homogenate was aseptically placed into a filter stomacher bag, to which 225 mL of sterile MHB was added and stomached for 3 min using a Colworth Stomacher 400 (Seward Ltd., UK). Ten milliliters of this homogenate were transferred into a sterile tube and incubated at 30°C for 18 h under constant agitation at 170 rpm on an orbital shaker. The enriched culture was considered the raw salmon microbiota. The initial bacterial load of this native salmon microbiota was determined using the spread plate method on MHA plates.

For the selective isolation of LAB and *B. thermosphacta* from the raw salmon microbiota, MRS agar and STAA agar base (supplemented with SR0151E) were used, respectively. The inoculated plates were incubated at 30°C for 72 h (LAB) or 25°C for 24 h ( *B. thermosphacta* ). After incubation, one single colony from MRS agar or STAA agar plates was streaked onto corresponding fresh agar plates, followed by re‐incubation for 72 h at 30°C or 24 h at 25°C for LAB and *B. thermosphacta*, respectively. Prior to colony polymerase chain reaction (PCR) characterization of isolates, as described below in Section 2.4.2.1, single well isolated colonies of LAB and *B. thermosphacta* were stained for Gram reaction and examined for catalase activity using 3% H_2_O_2_ and oxidase activity using oxidase strips as preliminary tests for bacterial characterization.

##### 2.4.2.1 Characterization of LAB and *B. thermosphacta* Isolates From Raw Salmon

Colony polymerase chain reaction (PCR) was performed on LAB and *B. thermosphacta* isolates obtained from raw salmon darnes, using the 16S rRNA sequencing method as outlined by O'Connor et al. (2024) with some modifications. Cells were lysed by transferring a single bacterial colony into 20 μL of PCR‐grade water (Thermo Scientific) and vortexed for 30 s. PCR was performed in a total volume of 50 μL using 25 μL of Phusion PCR master mix (Thermo Scientific), 10 μL GC enhancer, 8 μL of PCR‐grade water, 2.5 μL of the non‐specific primers 27F (5′‐AGAGTTTGATCATGGCTCA‐3′) and 1492R (5′‐TACGGTTACCTTGTTACGACTT‐3′) (primer stocks at 10 μM; Eurofin Genomics), and 2 μL of DNA template from lysed cells. Amplification was carried out with reaction conditions as follows: initial denaturation at 98°C for 30 s, followed by 35 cycles of 98°C for 10 s, annealing at 50°C for 30 s, and elongation at 72°C for 30 s with a final extension step at 72°C for 10 min. The resulting amplicons (5 μL) from each reaction were electrophoresed in a 1.5% (w/v) agarose gel. An OmegaFlour Plus Gel Documentation System (Aplegen, San Francisco, United States) was used for visualization. The PCR products were purified using the GeneJet PCR Purification Kit (Thermo Fisher Scientific, Waltham, MA, United States). DNA sequencing of the forward strand was performed by Genewiz (Leipzig, Germany). The resulting sequences were identified to species level (≥ 98%) using BLAST for comparison to sequences deposited in the GenBank database.

### 2.5 Antimicrobial Activity

#### 2.5.1 Minimum Inhibitory Concentration (MIC)

**FIGURE 1:**
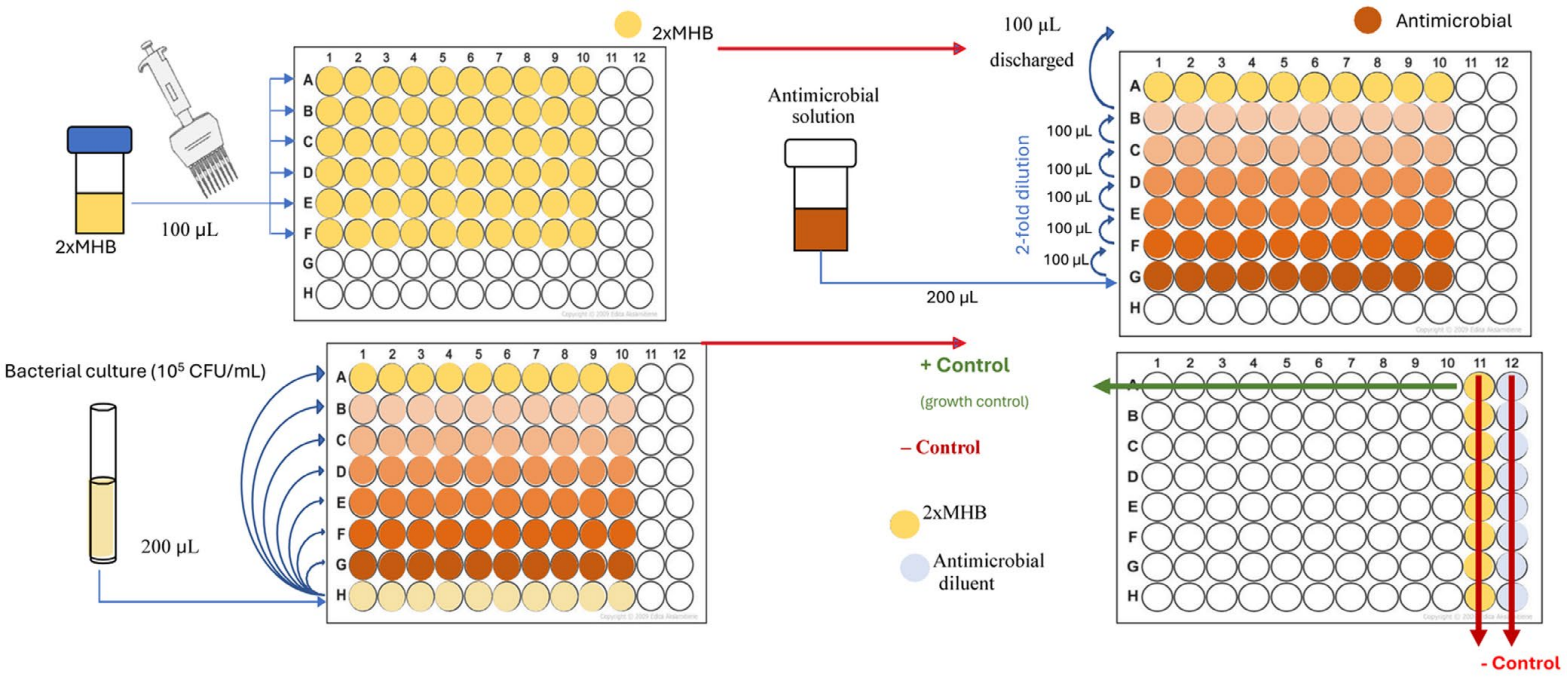
Schematic representation of the determination of MIC assay (adapted from Cruz‐Romero et al. (2013)).

AA of EOs (garlic, sage, marjoram, thyme, laurel, rosemary or oregano) emulsions, CS, ART and SUL were screened using the MIC assay according to the standard broth microdilution method described by Cruz‐Romero et al. (2013). As shown in Figure 1, a 96‐well flat‐bottom microplate (Sarstedt Inc., NC, USA) with an alpha alphanumeric layout (Columns 1–12, Rows A‐H) was used. A standardized bacterial inoculum (~105 CFU/mL) was prepared by diluting an overnight‐grown culture in sterile MHB and used within 15 mins. Two hundred μL of this suspension was added to Row H, Columns 1–11 and 200 μL of sterile double‐strength (2×) MHB was added to Column 12. Antimicrobial solution (200 μL) was added to Row G, then serially two‐fold diluted into corresponding wells containing 100 μL 2×MHB through Row B using a 12‐channel pipette. After mixing, 100 μL from Row B was discarded. Using a 12‐channel pipette, 15 μL of the mixed inoculum from Row H was transferred to corresponding wells in Rows A to G. Positive (Row A) and negative (Column 12) controls were included. The concentrations of 0.5% for EO emulsions and 0.1% for CS solution were determined through preliminary screening using varying concentrations of EOs (5%, 2%, 1%, 0.5%) and CS (0.5%, 0.25%, 0.1%), with 0.5% EO emulsions and 0.1% CS yielding optimal MIC values (in wells corresponding to Rows C or D) against all the bacteria.

The inoculated plates were incubated in a wet chamber for 24 h at 25°C ( *B. thermosphacta* ), 30°C ( *P. fluorescens* , LAB and salmon microbiota) or 37°C (*E. coli, S. aureus* and *L. innocua* ). Resazurin (0.015%) was added to wells A–F (30 μL per well), followed by 2 h of further incubation; wells showing no color change (resazurin remained blue, indicating no microbial growth) were scored as above the MIC value. The assay was independently repeated three times.

#### 2.5.2 Checkerboard Assay

**FIGURE 2:**
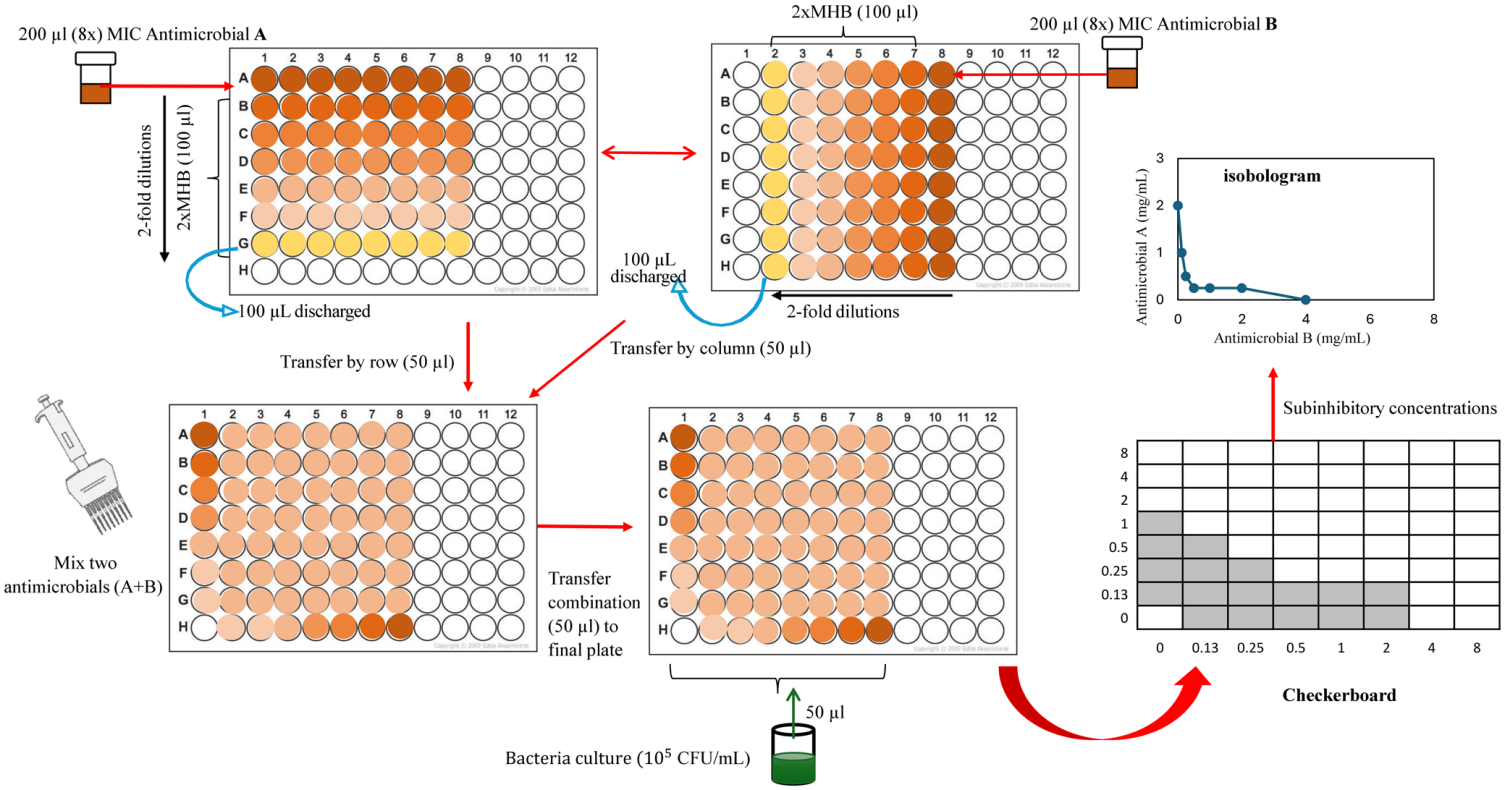
Schematic representation of antimicrobial combination using the checkerboard assay (adapted from Torres et al. (2018)).

To assess the synergistic potential of individual NAM in enhancing the antimicrobial spectrum through a combination approach, a checkerboard assay was employed, following the procedure outlined by Torres et al. (2018), with some modifications. The antimicrobial effect of 40 combinations, including EO–EO, EO–CS, EO–SUL and EO–ART systems, was evaluated. For each NAM tested, the initial concentration used was 8×MIC (eight times the MIC determined for each individual NAM as shown in Table 2). Bacterial cultures were grown overnight at the appropriate temperatures as outlined in Section 2.4 and adjusted to a final density of 10^5^ CFU mL^−1^ and used as inoculum within 15 min of preparation. As shown in Figure 2, In a 96‐well flat‐bottom microplate (Sarstedt Inc., NC, USA), 100 μL of 2×MHB was added to each well in rows B‐G, columns 1–8. Subsequently, 200 μL of antimicrobial ① at 8×MIC was added to wells in row A, columns 1–8. Using an eight‐channel pipette, a two‐fold serial dilution was performed by transferring 100 μL from row A into the corresponding wells of row B and continuing the dilution through row G. After thorough mixing at each step, 100 μL from row G was discarded to maintain equal volumes. In a second microplate, 100 μL of 2×MHB was added to each well in columns 2–7, rows A‐H. Then, 200 μL of antimicrobial ② at 8×MIC was added to wells in column 8, rows A–H. A two‐fold serial dilution was carried out horizontally by transferring 100 μL from column 8 into the corresponding wells of column 7 and continuing across to column 2. After mixing, 100 μL from column 2 was discarded. In a third microplate, 50 μL of each serially‐diluted antimicrobials (① and ②) was combined, with antimicrobial ① transferred along the rows and antimicrobial ② transferred along the columns to maintain combinations at sub‐inhibitory concentrations. The combined antimicrobials (① + ②) were mixed by pipetting 2–3 times in each well, after which 50 μL from each mixture was transferred into a fourth microplate. Finally, 50 μL of the bacterial suspension was added to each well to obtain the final assay volume. Column 1 and row H of the final plate contained only the serially diluted antimicrobial ① and antimicrobial ②, respectively, and appropriate positive and negative controls were included on the same plate. The inoculated plates were then incubated in a humid chamber for 24 h at 25°C ( *B. thermosphacta* ), 30°C ( *P. fluorescens* , LAB and salmon microbiota) or 37°C (*E. coli, S. aureus* and *L. innocua* ). Following incubation, 30 μL of resazurin dye (0.015% w/v) was added to each test well and plates were re‐incubated for an additional 2 h at the organism‐specific temperatures. The fractional inhibitory concentration (FIC) was recorded as the lowest concentration of the antimicrobial combination that prevented a color change in the resazurin indicator, signifying inhibition of metabolic activity. The assay was independently repeated three times, and for each experiment, a fractional inhibitory concentration index (FICI) was calculated according to the following equation:

FICI=FIC①+FIC②

$$\mathrm{FIC}①=\frac{\mathrm{MIC}\;\left(①+②\right)}{\mathrm{MIC}①}$$

$$\mathrm{FIC}②=\frac{\mathrm{MIC}\;\left(②+①\right)}{\mathrm{MIC}②}$$

where $\mathrm{FIC}①=\frac{\mathrm{MIC}\;\text{of antimicrobial}①\text{in the presence of antimicrobial}②}{\mathrm{MIC}\;\text{of antimicrobial}①\text{alone}}$ and $\mathrm{FIC}②=\frac{\mathrm{MIC}\;\text{of antimicrobial}②\text{in the presence of antimicrobial}①}{\mathrm{MIC}\;\text{of antimicrobial}②\text{alone}.}$

The FICI was used to assess the interaction of antimicrobial agents. A FICI ≤ 0.5 indicated synergistic effect, meaning the combination enhances AA. A FICI between > 0.5 and ≤ 1.0 was classified as additivity effect, suggesting the combined effect is equivalent to the sum of individual effects (Odds 2003).

#### 2.5.3 Time‐Kill Kinetic Assay

Checkerboard assays were first performed to evaluate potential interactions between the antimicrobials. As no significant differences were observed among strains within the same bacterial group (Gram‐positive, Gram‐negative or salmon microbiota), representative organisms were selected for subsequent kinetic analysis. *S. aureus* and *P. fluorescens* were chosen as the Gram‐positive and Gram‐negative models, respectively. Time–kill kinetics were conducted using the broth macrodilution method in accordance with CLSI guidelines (CLSI 1999; Israyilova et al. 2022), and the assay confirmed both the synergistic interaction of the antimicrobials and the bactericidal effect of the combined NAM. Bacterial strains were cultured overnight at the appropriate temperature as outlined in Section 2.4, to achieve concentration of 10^8^ CFU mL^−1^ and used as inoculum within 15 min of preparation. Selected antimicrobial combinations that showed synergistic effects (FICI ≤ 0.5) in the checkerboard assay were tested at 0.5×MIC, 1×MIC and 2×MIC concentrations. In a sterile 200 mL Erlenmeyer flasks, a 0.5 mL aliquot of the overnight‐grown bacterial culture (*P. fluorescens or S. aureus*) was added to 50 mL of antimicrobial combinations prepared at 0.5×MIC, 1×MICand 2×MIC in sterile MHB, yielding a final cell density of $10^{6}$ CFU mL^−1^ in the solution. The solutions were then incubated with constant shaking (170 rpm) at 30°C for *P. fluorescens* or 37°C for *S. aureus* .

The microbial load at designated time intervals (0, 3, 6, 9, 12, 24 and 36 h) was then determined using the pour plate method. At each time point, 1 mL aliquots were aseptically withdrawn, serially diluted in sterile MRD and 1 mL of an appropriate 10‐fold dilution was inoculated into 15 mL of molten MHA (45°C). The plates were incubated at 30°C for *P. fluorescens* or 37°C for *S. aureus* , with CFU/mL were enumerated after 24 h of incubation. Control tests (solutions without antimicrobials) were also included and the experiment was repeated on three independent occasions. Bacterial viability was assessed by plotting CFU/mL against sampling time to evaluate growth kinetics. Antimicrobial combinations were considered bactericidal if they resulted in a ≥ 3 log_10_ reduction in CFU compared to the initial bacterial load of the inoculum, corresponding to 99.9% bacterial killing. In contrast, bacteriostatic activity was defined as if it maintained the original bacterial count or achieving a reduction of less than 3 log_10_ CFU/mL (Israyilova et al. 2022).

### 2.6 Statistical Analysis

Data were recorded as mean value ± standard error (SE) from three independent measurements (*n* = 6). SPSS software was utilized for data analysis. One‐way ANOVA followed by Tukey's post hoc test was performed to determine the levels of differences, and a confidence level of *p* < 0.05 was considered statistically significant.

## 3 Results and Discussion

### 3.1 Particle Size and Polydispersity Index (PDI) of EO Emulsions

Particle size and PDI measurements provide important insight into the stability and potential antimicrobial performance of EO emulsions (He et al. 2022; Kamau et al. 2026). Overall, thyme and oregano EOs produced the smallest (*p* < 0.05) particle size compared to rosemary, sage, marjoram, laurel and garlic EO emulsions (Table 1). Of all EOs, garlic EO produced the biggest (*p* < 005) particle size while no significance difference on the particle size (*p* > 0.05) was noticed between emulsions obtained using thyme or oregano EOs. PDI, a dimensionless measure of the homogeneity of the particle size distribution and stability of the droplet size calculated from the cumulant analysis (Sullivan et al. 2017) indicated that the emulsions obtained using thyme and oregano EOs also exhibited significantly (*p* < 0.05) lower PDI values (0.1), indicating a more homogeneous droplet size distributions, suggesting good physical stability when compared to marjoram, laurel and garlic EO emulsions. Similar results have been reported for thyme and oregano emulsions prepared using ultrasonication (Al‐Asmari et al. 2024), reflecting their high emulsification efficiency. In this study, the particle size of the EO emulsion may have been influenced by both the emulsification method and the chemical composition of the EOs, as previous reports indicate that chemical composition, emulsification method and physicochemical properties of the EO can significantly affect emulsion particle size (Al‐Asmari et al. 2024; Đurović et al. 2022; Kamau et al. 2026; Tavares et al. 2021; Zhang and Wang 2019). Compared to thyme and oregano EOs; rosemary, sage, marjoram, laurel and garlic EOs contain lower levels of phenolic compounds (Table S1), which may affect the particle size distribution of the EO emulsion. In contrast, marjoram, laurel and garlic EOs contain higher proportions of highly hydrophobic or viscous constituents such as eugenol derivatives, monoterpenes, sabinene and organo‐sulfur compounds (Table S1) which reportedly increase interfacial tension that can hinder efficient droplet breakup during emulsification (Đurović et al. 2022). As a result, these oils tend to form emulsions with larger droplet sizes. As shown in this study, marjoram, laurel and garlic EOs produced emulsions with larger droplets (256–293 nm) and higher PDIs (0.2), reflecting reduced emulsification efficiency and greater polydispersity. In addition, the lower interfacial adsorption kinetics of these bulky or viscous molecules reduce their ability to rapidly adsorb onto newly formed droplet surfaces, thereby limiting stabilization efficiency and promoting coalescence during emulsification (Đurović et al. 2022). The emulsification process is further influenced by physicochemical parameters such as the viscosity ratio between the dispersed oil phase and the continuous aqueous phase; the closer this ratio is to unity, the more efficient the mechanical emulsification process becomes (Donsì and Ferrari 2016). In this context, the viscosity differences among the investigated EOs may have contributed to the observed emulsion behavior. Notably, garlic EO is known to contain high levels of diallyl sulfides and other organo‐sulfur compounds (Table S1), which impart a markedly higher viscosity compared with other culinary EOs (Shang et al. 2019). Consistent with these reports, garlic EO emulsion in the present study appeared visually more viscous, which likely impeded droplet disruption and contributed to the formation of larger droplet sizes.

**TABLE 1:** Particle size distribution and polydispersity index (PDI) of oregano, thyme, marjoram, sage, rosemary, laurel and garlic EO emulsions.*

Emulsion	Particle size (nm)	PDI
Thyme EO	162.3 ± 4.76^a^	0.1 ± 0.02^a^
Oregano EO	162.1 ± 7.57^a^	0.1 ± 0.03^a^
Rosemary EO	182.0 ± 3.97^b^	0.1 ± 0.03^a^
Sage EO	184.6 ± 1.02^b^	0.1 ± 0.00^a^
Marjoram EO	256.3 ± 11.52^c^	0.2 ± 0.05^b^
Laurel EO	261.6 ± 19.81^c^	0.2 ± 0.01^b^
Garlic EO	293.0 ± 5.89^d^	0.2 ± 0.02^b^

*Note:* Different superscript letters in the same column indicate significant differences (*p* < 0.05).

* All values are means ± SE of triplicate measurements from two independent experiments (*n* = 6).

### 3.2 Characterization of LAB and *B. thermosphacta* Strains Isolated From Raw Salmon

The LAB and *B. thermosphacta* isolated from salmon microbiota were identified using 16S rRNA gene sequencing, a widely applied molecular tool in bacterial systematics due to the conserved nature of the 16S rRNA gene and its suitability for resolving phylogenetic relationships (Kadir 2025). The sequencing of the LAB isolates from raw salmon microbiota showed a very high (> 99%) similarity to members of the *Lactobacillus casei/paracasei* group, with BLAST results showing percent identities exceeding 99%, extremely low E‐values and strong query coverage across multiple reference sequences (Table S2). This finding is in agreement with earlier reports documenting the presence of *Lactobacillus* species in raw, processed or fermented fish products, showing that LAB form part of fish natural microbiota (Alonso et al. 2019; Govindaraj et al. 2021; Ringø et al. 2018). Their occurrence in fish has been attributed to the capacity of LAB to grow in protein‐rich substrates, moderately saline and low‐temperature environments which are characteristic of fish matrices (Ayivi and Ibrahim 2022). Additionally, LAB are facultative anaerobes, enabling them to grow under both aerobic and anaerobic conditions, which enhances their survival during processing and storage (Mokoena 2017). Other factors that also influence LAB growth include pH, water activity, availability of fermentable carbohydrates, temperature fluctuations and competition with other microorganisms (Ayivi and Ibrahim 2022).

The sequencing analysis of the *B. thermosphacta* isolated from raw salmon also showed > 99% identity to multiple reference *B. thermosphacta* strains (Table S3), confirming its taxonomic assignment and underscoring its relevance in fish spoilage, as *B. thermosphacta* is a psychrotrophic species frequently implicated in the deterioration of chilled meat and fish products (Bouju‐Albert et al. 2021). *B. thermosphacta* is well known for its ability to proliferate at low temperatures and metabolize amino acids and other substrates in protein‐dense environments, producing volatile compounds associated with off‐odors and sensory decline of stored packaged fish products. Its recurrent detection in fish and fish products, particularly during prolonged cold storage, has been widely reported, with its dominance influenced by storage atmosphere and interactions with competing microbiota (Bouju‐Albert et al. 2021; Illikoud et al. 2019). Overall, the application of 16S rRNA gene sequencing provided robust confirmation that both LAB and *B. thermosphacta* isolated from raw salmon microbiota were successfully identified, indicating that both LAB and *B. thermosphacta* were present in raw salmon samples, supporting the fact that these organisms form part of the native microbial community associated with fish.

### 3.3 Antimicrobial Activity

#### 3.3.1 Minimum Inhibitory Concentration

Initial screening of the NAM (CS, SUL, ART and EOs; Oregano (OEO), Thyme (TEO), Garlic (GEO), Sage (SEO), Marjoram (MEO), Laurel (LEO) and Rosemary (REO)), showed that CS exhibited significantly (*p* < 0.05) higher AA (MIC of 0.125 mg/mL) against all Gram‐positive (*S. aureus, L. innocua*, *B. thermosphacta* and LAB), Gram‐negative bacteria ( *E. coli* , *P. fluorescens* ) and salmon microbiota as compared to all other antimicrobials tested (Table 2). Similar MIC values against a broad spectrum of Gram‐positive and Gram‐negative bacteria for low‐molecular weight CS were reported by Shanmugam et al. (2016). The high antimicrobial effectiveness of low‐molecular weight CS has been attributed to its enhanced ability to penetrate the bacterial cell wall and membrane, thereby disrupting essential cellular processes such as RNA and protein synthesis and ultimately leading to cell death (Chandrasekaran et al. 2020; Ke et al. 2021; Yan et al. 2021). Among the EOs examined, OEO showed the highest (*p* < 0.05) AA against all tested Gram‐positive and Gram‐negative bacteria, as well as salmon microbiota, as indicated by its lower MIC value (0.313 mg/mL). The high AA of OEO may be attributed to higher concentration of phenolic components, particularly carvacrol that was dominant (Table S1) as well as smaller particle size (~162 nm) as shown in Table 1. Reduced droplet size and lower PDI are known to enhance the surface area and facilitate closer interactions between EO droplets and bacterial cells, thereby promoting penetration into the cell membrane especially the lipid components, thereby maximizing antimicrobial potential (Kamau et al. 2026; Mahmud et al. 2024; McClements et al. 2021). In contrast, REO, LEO and MEO EOs showed comparable antimicrobial activities (MIC: 0.625 mg/mL) across Gram‐positive and Gram‐negative bacteria and salmon microbiota, with no statistically significant (*p* > 0.05) differences observed among the tested microorganisms. This uniformity may be related to their relatively moderate content of phenolic compounds and the predominance of less potent terpenoids (such as 1,8‐cineole, Terpinen‐4‐ol, α‐pinene and camphor) (Table S1), which generally exhibit weaker membrane disruptive effects compared with highly active phenolics such as carvacrol and thymol, resulting in reduced selectivity between bacterial groups and more uniform antimicrobial responses across different microorganisms (Ben Amor et al. 2021). For TEO, SEO and GEO EOs, Gram‐negative bacteria and salmon microbiota were more resistant (MIC 0.625 mg/mL) to those antimicrobials compared to Gram‐positive bacteria (MIC 0.313 mg/mL). This pattern is consistent with previous reports indicating a higher intrinsic resistance of Gram‐negative bacteria to EOs, largely attributed to structural differences in the cell envelope (Semeniuc et al. 2017; Sharifi‐Rad et al. 2018; Speranza et al. 2023). Generally, compared to our findings, variability in the reported MIC values of EOs, including TEO (MIC 0.32 mg/mL) and OEO (MIC 0.64 mg/mL) (Boskovic et al. 2015), REO (MIC 0.07 mg/mL), LEO (MIC 0.8 mg/mL) and MEO (MIC 0.4 mg/mL) (Ben Amor et al. 2021; El‐Shenawy et al. 2015; Thielmann et al. 2019), SEO (MIC 0.09 mg/mL) (Ben Amor et al. 2021) and GEO (MIC 0.1–0.5 mg/mL) (Sarangi et al. 2024), against a wide range of Gram‐positive and Gram‐negative bacteria have been reported. Such discrepancies may, among other factors, be attributed to variations in chemical composition, intrinsic and environmental influences (Kamau et al. 2025), as well as differences in particle size, which is itself affected by the emulsion preparation method. Our previous work demonstrated that the emulsification method has a direct impact on the AA of EOs. In particular, ultrasonication produced high AA, which correlated with smaller droplet sizes, narrower PDI values and improved emulsion stability (Kamau et al. 2026).

**TABLE 2:** Minimum inhibitory concentration (MIC) values (mg/mL) for CS, SUL, ART and EOs (Oregano (OEO), Thyme (TEO), Garlic (GEO), Sage (SEO), Marjoram (MEO), Laurel (LEO) and Rosemary (REO)).*

Bacteria	MIC (mg/mL)									
	CS	OEO	TEO	GEO	SEO	LEO	MEO	REO	SUL	ART
*S. aureus*	0.125^a^	0.313^b^	0.313^b^	0.313^b^	0.313^b^	0.625^c^	0.625^c^	0.625^c^	3.750^d^	25.000^e^
*L. innocua*	0.125^a^	0.313^b^	0.313^b^	0.313^b^	0.313^b^	0.625^c^	0.625^c^	0.625^c^	3.750^d^	25.000^e^
*B. thermosphacta*	0.125^a^	0.313^b^	0.313^b^	0.313^b^	0.313^b^	0.625^c^	0.625^c^	0.625^c^	3.750^d^	25.000^e^
LAB	0.125^a^	0.313^b^	0.313^b^	0.313^b^	0.313^b^	0.625^c^	0.625^c^	0.625^c^	3.750^d^	25.000^e^
*E. coli*	0.125^a^	0.313^b^	0.625^c^	0.625^c^	0.625^c^	0.625^c^	0.625^c^	0.625^c^	3.750^d^	50.000^e^
*P. fluorescens*	0.125^a^	0.313^b^	0.625^c^	0.625^c^	0.625^c^	0.625^c^	0.625^c^	0.625^c^	3.750^d^	50.000^e^
Salmon microbiota	0.125^a^	0.313^b^	0.625^c^	0.625^c^	0.625^c^	0.625^c^	0.625^c^	0.625^c^	3.750^d^	50.000^e^

*Note:* Different superscript letters in the same row indicate significant differences (*p* < 0.05).

* All values are means of triplicate measurements from two independent experiments (*n* = 6).

Among the commercially available mixtures of weak organic acids examined, SUL demonstrated significantly (*p* < 0.05) higher AA against the tested Gram‐positive and Gram‐negative bacteria, as well as salmon microbiota, as indicated by its lower MIC value (3.75 mg/mL). This enhanced AA even against the complex salmon microbiota is likely linked to the high lactic acid content of SUL, which was reported to disrupt bacterial metabolism through intracellular acidification (Stanojević‐Nikolić et al. 2016). With respect to the efficacy of ART against pure cultures of Gram‐negative bacteria and salmon microbiota, no significant (*p* > 0.05) differences were noticed in AA. The results indicated that Gram‐negative and salmon microbiota were more (*p* < 0.05) resistant than Gram‐positive bacteria (Table 2). The generally higher MIC values observed for ART may be attributed to its dilution to 20% prior to use, whereas SUL was diluted only to 3%. Further, the AA of ART is largely attributable to the combined effects of its organic acid constituents (sodium diacetate, lactic acid, acetic acid, and citric acid), which collectively lower extracellular pH, disrupt membrane integrity, and accumulate intracellularly as undissociated acids that interfere with metabolic homeostasis (Cruz‐Romero et al. 2013; Kamau et al. 2025). Previous studies have reported that the effectiveness of weak organic acids can vary according to their molecular weight; compared to organic acid solubilates in ART, LA has smaller undissociated molecules that can penetrate bacterial cells more readily and alter intracellular pH (Stanojević‐Nikolić et al. 2016). The differences in molecular weight may help explain the greater bacterial sensitivity observed for SUL compared with ART, as SUL contains lactic acid as its sole active component.

#### 3.3.2 Checkerboard Assay

##### 3.3.2.1 CS and EOs Combination

Combinations of CS with various EOs significantly (*p* < 0.05) reduced the antimicrobial concentrations required to inhibit bacterial growth. Among these, the combination CS + GEO exhibited the most pronounced synergistic effect (FICI = 0.19) against all tested Gram‐positive bacteria, resulting in a 16‐fold reduction in the MIC of GEO and an 8‐fold reduction of CS, respectively (Table 3). These strong synergistic interaction between CS and GEO, can be attributed to the high complementarity between their antimicrobial mechanisms, coupled with the inherent susceptibility of Gram‐positive bacteria to EOs (da Silva et al. 2023; Kamau et al. 2025). GEO contains reactive organo‐sulfur compounds such as allicin, diallyl sulfide and diallyl disulfide, which have been reported to exert potent intracellular antimicrobial effects by reacting with thiol‐containing enzymes, disrupting redox balance and inhibiting DNA and protein synthesis (Sarangi et al. 2024). These intracellular targets differ markedly from the membrane‐focused mode of action typical of many other EOs. CS's electrostatic interaction with negatively charged microbial surfaces may have increased membrane permeability, thereby facilitating the penetration of allicin and related sulfur compounds into the cytoplasm resulting into multi‐targeted antimicrobial membrane destabilization that overwhelms bacterial defense systems more effectively than either agent alone (Chandrasekaran et al. 2020; Ke et al. 2021; Yan et al. 2021). Synergistic effects were also consistently observed when CS was combined with MEO, LEO or REO (FICI = 0.5), each yielding a 4‐fold decrease in the MIC values of CS or the corresponding EO across all tested bacteria. This enhanced activity reflects the complementary mechanisms that emerge when CS is used in combination with EOs. CS not only maintains its broad‐spectrum antimicrobial properties but also serves as an effective synergistic partner by stabilizing EO constituents through interactions at the cell surface. Its cationic amino groups enable strong electrostatic binding to negatively charged microbial membranes, improving the localisation and efficacy of the hydrophobic EO component (Kamau et al. 2025). The combined action of CS and EOs can generate a semi‐permeable antimicrobial matrix that may disrupt respiration, alters membrane ion permeability, particularly for hydrogen and potassium ions, and interferes with enzyme systems and genetic material, ultimately leading to oxidative damage and cell death (Raphaël and Meimandipour 2017). This multi‐targeted mode of action can enhance antimicrobial efficacy at lower dosages and, as a result, help overcome the sensory limitations typically associated with the use of EOs as antimicrobials, since relatively high concentrations are otherwise required to achieve effective inhibition, thereby restricting their practical application (Kamau et al. 2025; Munhuweyi et al. 2017). Similar to the findings of this study, synergistic effect between CS and EOs such as rosemary (Elsherif et al. 2024), garlic (Molaee Aghaee et al. 2016), oregano and thyme (Munhuweyi et al. 2017; Raphaël and Meimandipour 2017) have been reported against foodborne pathogens and spoilage organisms. While synergistic effect was observed when CS was combined with MEO, LEO or REO, the combination of CS with SEO or TEO produced an additive (FICI = 0.75) effect against Gram‐positive bacteria and a synergistic (FICI = 0.5) effect against Gram‐negative bacteria or salmon microbiota. Independent of the bacterial type tested (Gram‐positive, Gram‐negative bacteria or salmon microbiota), the combination of CS and OEO showed an additive (FICI = 0.75) effect. This additive effect may be attributed to the strong antimicrobial activity of both agents (CS and OEO), as indicated by their low MIC values. When two highly active antimicrobials are combined, a ceiling effect often occurs, leaving limited scope for further MIC reduction, a pattern frequently reported for combinations involving highly active/effective antimicrobials (Bassolé and Juliani 2012; Hyldgaard et al. 2012). Although a synergistic effect was not noticed, nevertheless, the antimicrobial combination of CS and OEO resulted in significant reductions in individual antimicrobial concentration requirements, lowering the MICs of CS by 4‐fold and OEO by 2‐fold compared to the MICs of individual antimicrobials.

**TABLE 3:** Fractional inhibition concentration index (FICI) values for combinations of Chitosan (CS) with Oregano (OEO), Thyme (TEO), Garlic (GEO), Sage (SEO), Marjoram (MEO), Laurel (LEO), Rosemary (REO) EOs.*

Bacteria	Combinations	MIC alone (mg/mL)		MIC in combination (mg/mL)		FIC		FICI	Effect
		CS	EO	CS	EO	CS	EO		
*S. aureus*	CS + OEO	0.125	0.313	0.031	0.156	0.25	0.50	0.75^c^	Additivity
	CS + TEO	0.125	0.313	0.031	0.156	0.25	0.50	0.75^c^	Additivity
	CS + SEO	0.125	0.313	0.031	0.156	0.25	0.50	0.75^c^	Additivity
	CS + GEO	0.125	0.313	0.008	0.039	0.06	0.12	0.19^a^	Synergistic
	CS + MEO	0.125	0.625	0.031	0.156	0.25	0.25	0.50^b^	Synergistic
	CS + LEO	0.125	0.625	0.031	0.156	0.25	0.25	0.50^b^	Synergistic
	CS + REO	0.125	0.625	0.031	0.156	0.25	0.25	0.50^b^	Synergistic
*L. innocua*	CS + OEO	0.125	0.313	0.031	0.156	0.25	0.50	0.75^c^	Additivity
	CS + TEO	0.125	0.313	0.031	0.156	0.25	0.50	0.75^c^	Additivity
	CS + SEO	0.125	0.313	0.031	0.156	0.25	0.50	0.75^c^	Additivity
	CS + GEO	0.125	0.313	0.008	0.039	0.06	0.12	0.19^a^	Synergistic
	CS + MEO	0.125	0.625	0.031	0.156	0.25	0.25	0.50^b^	Synergistic
	CS + LEO	0.125	0.625	0.031	0.156	0.25	0.25	0.50^b^	Synergistic
	CS + REO	0.125	0.625	0.031	0.156	0.25	0.25	0.50^b^	Synergistic
*B. thermosphacta*	CS + OEO	0.125	0.313	0.031	0.156	0.25	0.50	0.75^c^	Additivity
	CS + TEO	0.125	0.313	0.031	0.156	0.25	0.50	0.75^c^	Additivity
	CS + SEO	0.125	0.313	0.031	0.156	0.25	0.50	0.75^c^	Additivity
	CS + GEO	0.125	0.313	0.008	0.039	0.06	0.12	0.19^a^	Synergistic
	CS + MEO	0.125	0.625	0.031	0.156	0.25	0.25	0.50^b^	Synergistic
	CS + LEO	0.125	0.625	0.031	0.156	0.25	0.25	0.50^b^	Synergistic
	CS + REO	0.125	0.625	0.031	0.156	0.25	0.25	0.50^b^	Synergistic
LAB	CS + OEO	0.125	0.313	0.031	0.156	0.25	0.50	0.75^c^	Additivity
	CS + TEO	0.125	0.313	0.031	0.156	0.25	0.50	0.75^c^	Additivity
	CS + SEO	0.125	0.313	0.031	0.156	0.25	0.50	0.75^c^	Additivity
	CS + GEO	0.125	0.313	0.008	0.039	0.06	0.12	0.19^a^	Synergistic
	CS + MEO	0.125	0.625	0.031	0.156	0.25	0.25	0.50^b^	Synergistic
	CS + LEO	0.125	0.625	0.031	0.156	0.25	0.25	0.50^b^	Synergistic
	CS + REO	0.125	0.625	0.031	0.156	0.25	0.25	0.50^b^	Synergistic
*E. coli*	CS + OEO	0.125	0.313	0.031	0.156	0.25	0.50	0.75^c^	Additivity
	CS + TEO	0.125	0.625	0.031	0.156	0.25	0.25	0.50^b^	Synergistic
	CS + GEO	0.125	0.625	0.031	0.156	0.25	0.25	0.50^b^	Synergistic
	CS + SEO	0.125	0.625	0.031	0.156	0.25	0.25	0.50^b^	Synergistic
	CS + MEO	0.125	0.625	0.031	0.156	0.25	0.25	0.50^b^	Synergistic
	CS + LEO	0.125	0.625	0.031	0.156	0.25	0.25	0.50^b^	Synergistic
	CS + REO	0.125	0.625	0.031	0.156	0.25	0.25	0.50^b^	Synergistic
*P. fluorescens*	CS + OEO	0.125	0.313	0.031	0.156	0.25	0.50	0.75^c^	Additivity
	CS + TEO	0.125	0.625	0.031	0.156	0.25	0.25	0.50^b^	Synergistic
	CS + GEO	0.125	0.625	0.031	0.156	0.25	0.25	0.50^b^	Synergistic
	CS + SEO	0.125	0.625	0.031	0.156	0.25	0.25	0.50^b^	Synergistic
	CS + MEO	0.125	0.625	0.031	0.156	0.25	0.25	0.50^b^	Synergistic
	CS + LEO	0.125	0.625	0.031	0.156	0.25	0.25	0.50^b^	Synergistic
	CS + REO	0.125	0.625	0.031	0.156	0.25	0.25	0.50^b^	Synergistic
Salmon microbiota	CS + OEO	0.125	0.313	0.031	0.156	0.25	0.50	0.75^c^	Additivity
	CS + TEO	0.125	0.625	0.031	0.156	0.25	0.25	0.50^b^	Synergistic
	CS + GEO	0.125	0.625	0.031	0.156	0.25	0.25	0.50^b^	Synergistic
	CS + SEO	0.125	0.625	0.031	0.156	0.25	0.25	0.50^b^	Synergistic
	CS + MEO	0.125	0.625	0.031	0.156	0.25	0.25	0.50^b^	Synergistic
	CS + LEO	0.125	0.625	0.031	0.156	0.25	0.25	0.50^b^	Synergistic
	CS + REO	0.125	0.625	0.031	0.156	0.25	0.25	0.50^b^	Synergistic

*Note:* Different superscript letters in FICI indicate statistically significant differences (*p* < 0.05), based on comparisons performed across all combinations and all bacteria tested.

* All values are means of triplicate measurements from two independent experiments (*n* = 6).

##### 3.3.2.2 Combination of Commercially Available Mixtures of Weak Organic Acids (SUL&ART) and EOs

Combinations of ART with all EOs (OEO, TEO, GEO, SEO, MEO, LEO or REO) significantly (*p* < 0.05) reduced the antimicrobial concentrations of each antimicrobial required to inhibit bacterial growth. Independent of the bacterial type tested (Gram‐positive, Gram‐negative bacteria or salmon microbiota), the combination of ART and GEO showed the strongest synergistic (FICI = 0.25) effect, resulting in an 8‐fold reduction in the MICs of both antimicrobials when used in combination compared to the MIC when each antimicrobial is used alone. This synergistic effect between CS and GEO reflects the distinct and highly complementary antimicrobial mechanisms of these antimicrobials as organo‐sulfur compounds present in GEO act predominantly on intracellular targets, in contrast to phenolic‐rich EOs that primarily exert membrane‐level effects (Nazzaro et al. 2013). A similar synergistic (FICI = 0.25) effect was observed when ART was combined with OEO, TEO or SEO against Gram‐negative bacteria and the salmon microbiota, as well as when ART was combined with MEO, LEO or REO against Gram‐positive bacteria (Table 4). Notably, the ART + MEO, ART + LEO and ART + REO combinations produced a 4‐fold reduction in the MIC of ART and a 31‐fold reduction in the MIC of the respective EOs, whereas ART + OEO, ART + TEO and ART + SEO resulted in an 8‐fold reduction in the MIC of both antimicrobials. These findings indicate that equivalent antimicrobial inhibition can be achieved at significantly lower concentrations than when each compound is applied individually. Although the AA of ART in combination with EOs has not been previously reported, the synergistic effect observed here is likely attributable to the presence of organic acid solubilates in ART, including lactic acid, acetic acid and sodium diacetate, which can act complementarily with EOs due to their distinct modes of antimicrobial action. For example, the synergistic effect between oregano EO and acetic acid or lactic acid has been reported against pathogenic *S. aureus* , *E. coli* and *L. monocytogenes* (Ijabadeniyi et al. 2020). As such, weak organic acids penetrate bacterial membranes in their undissociated form and dissociate intracellularly, lowering cytoplasmic pH and disrupting proton‐anion balance, impairing nutrient transport and ultimately leading to cell death (Kamau et al. 2025), while phenolic compounds present in EOs can destabilize bacterial membranes, increase permeability and induce leakage of cellular contents, thereby enhancing bacterial susceptibility to acidic environments (Gómez‐García et al. 2019; Sorathiya et al. 2025). Moreover, under acidic conditions, EO hydrophobicity and partitioning into lipid bilayers are further enhanced, amplifying their disruptive action on bacterial cells (de Barros et al. 2012). Therefore, the complementary antimicrobial mechanisms of weak organic acids and EOs have the potential to form multi‐mechanistic antimicrobial combinations that allow for reduced dosages of individual agents, thereby minimizing sensory impacts while enhancing microbial safety in food systems.

**TABLE 4:** Fractional inhibition concentration index (FICI) values for combinations of Oregano (OEO), Thyme (TEO), Garlic (GEO), Sage (SEO), Marjoram (MEO), Laurel (LEO), Rosemary (REO) EOs and ART.*

Bacteria	Combinations	MIC alone (mg/mL)		MIC in combination (mg/mL)		FIC		FICI	Effect
		ART	EO	ART	EO	ART	EO		
*S. aureus*	ART+OEO	25.000	0.313	6.250	0.040	0.25	0.13	0.38^c^	Synergistic
	ART+TEO	25.000	0.313	6.250	0.040	0.25	0.13	0.38^c^	Synergistic
	ART+GEO	25.000	0.313	3.130	0.040	0.13	0.13	0.25^a^	Synergistic
	ART+SEO	25.000	0.313	6.250	0.020	0.25	0.06	0.31^b^	Synergistic
	ART+MEO	25.000	0.625	6.250	0.020	0.25	0.03	0.28^a^	Synergistic
	ART+LEO	25.000	0.625	6.250	0.020	0.25	0.03	0.28^a^	Synergistic
	ART+REO	25.000	0.625	6.250	0.020	0.25	0.03	0.28^a^	Synergistic
*L. innocua*	ART+OEO	25.000	0.313	6.250	0.040	0.25	0.13	0.38^c^	Synergistic
	ART+TEO	25.000	0.313	6.250	0.040	0.25	0.13	0.38^c^	Synergistic
	ART+GEO	25.000	0.313	3.130	0.040	0.13	0.13	0.25^a^	Synergistic
	ART+SEO	25.000	0.313	6.250	0.020	0.25	0.06	0.31^b^	Synergistic
	ART+MEO	25.000	0.625	6.250	0.020	0.25	0.03	0.28^a^	Synergistic
	ART+LEO	25.000	0.625	6.250	0.020	0.25	0.03	0.28^a^	Synergistic
	ART+REO	25.000	0.625	6.250	0.020	0.25	0.03	0.28^a^	Synergistic
*B. thermosphacta*	ART+OEO	25.000	0.313	6.250	0.040	0.25	0.13	0.38^c^	Synergistic
	ART+TEO	25.000	0.313	6.250	0.040	0.25	0.13	0.38^c^	Synergistic
	ART+GEO	25.000	0.313	3.130	0.040	0.13	0.13	0.25^a^	Synergistic
	ART+SEO	25.000	0.313	6.250	0.020	0.25	0.06	0.31^b^	Synergistic
	ART+MEO	25.000	0.625	6.250	0.020	0.25	0.03	0.28^a^	Synergistic
	ART+LEO	25.000	0.625	6.250	0.020	0.25	0.03	0.28^a^	Synergistic
	ART+REO	25.000	0.625	6.250	0.020	0.25	0.03	0.28^a^	Synergistic
LAB	ART+OEO	25.000	0.313	6.250	0.040	0.25	0.13	0.38^c^	Synergistic
	ART+TEO	25.000	0.313	6.250	0.040	0.25	0.13	0.38^c^	Synergistic
	ART+GEO	25.000	0.313	3.130	0.040	0.13	0.13	0.25^a^	Synergistic
	ART+SEO	25.000	0.313	6.250	0.020	0.25	0.06	0.31^b^	Synergistic
	ART+MEO	25.000	0.625	6.250	0.020	0.25	0.03	0.28^a^	Synergistic
	ART+LEO	25.000	0.625	6.250	0.020	0.25	0.03	0.28^a^	Synergistic
	ART+REO	25.000	0.625	6.250	0.020	0.25	0.03	0.28^a^	Synergistic
*E. coli*	ART+OEO	50.000	0.313	6.250	0.040	0.13	0.13	0.25^a^	Synergistic
	ART+TEO	50.000	0.625	6.250	0.080	0.13	0.13	0.25^a^	Synergistic
	ART+GEO	50.000	0.625	6.250	0.080	0.13	0.13	0.25^a^	Synergistic
	ART+SEO	50.000	0.625	6.250	0.080	0.13	0.13	0.25^a^	Synergistic
	ART+MEO	50.000	0.625	12.500	0.160	0.25	0.25	0.50^d^	Synergistic
	ART+LEO	50.000	0.625	12.500	0.160	0.25	0.25	0.50^d^	Synergistic
	ART+REO	50.000	0.625	12.500	0.160	0.25	0.25	0.50^d^	Synergistic
*P. fluorescens*	ART+OEO	50.000	0.313	6.250	0.040	0.13	0.13	0.25^a^	Synergistic
	ART+TEO	50.000	0.625	6.250	0.080	0.13	0.13	0.25^a^	Synergistic
	ART+GEO	50.000	0.625	6.250	0.080	0.13	0.13	0.25^a^	Synergistic
	ART+SEO	50.000	0.625	6.250	0.080	0.13	0.13	0.25^a^	Synergistic
	ART+MEO	50.000	0.625	12.500	0.160	0.25	0.25	0.50^d^	Synergistic
	ART+LEO	50.000	0.625	12.500	0.160	0.25	0.25	0.50^d^	Synergistic
	ART+REO	50.000	0.625	12.500	0.160	0.25	0.25	0.50^d^	Synergistic
	ART+OEO	50.000	0.313	6.250	0.040	0.13	0.13	0.25^a^	Synergistic
Salmon microbiota	ART+TEO	50.000	0.625	6.250	0.080	0.13	0.13	0.25^a^	Synergistic
	ART+GEO	50.000	0.625	6.250	0.080	0.13	0.13	0.25^a^	Synergistic
	ART+SEO	50.000	0.625	6.250	0.080	0.13	0.13	0.25^a^	Synergistic
	ART+MEO	50.000	0.625	12.500	0.160	0.25	0.25	0.50^d^	Synergistic
	ART+LEO	50.000	0.625	12.500	0.160	0.25	0.25	0.50^d^	Synergistic
	ART+REO	50.000	0.625	12.500	0.160	0.25	0.25	0.50^d^	Synergistic

*Note:* Different superscript letters in FICI indicate statistically significant differences (*p* < 0.05), based on comparisons performed across all combinations and all bacteria tested.

* All values are means of triplicate measurements from two independent experiments (*n* = 6).

Similarly, combinations of SUL with all EOs (OEO, TEO, GEO, SEO, MEO, LEO or REO) significantly (*p* < 0.05) reduced the antimicrobial concentrations required to inhibit bacterial growth. Overall, the combinations of SUL with GEO or with SEO showed the strongest synergistic effect against all Gram‐positive bacteria tested ( *S. aureus* , *L. innocua* , *B. thermosphacta* , and LAB), as indicated by a low FICI value (FICI = 0.19). This synergistic effect resulted in a 17‐fold reduction in the MIC of SUL and an 8‐fold reduction in the MIC of GEO or SEO, compared to MICs of the antimicrobials when used alone (Table 5). This pronounced dose reduction effect may be attributed to the complementary antimicrobial mechanisms of lactic acid (a major component of SUL), which induce acid stress, increase membrane permeability, and compromise cell wall integrity of bacteria, thereby facilitating the enhanced diffusion of hydrophobic EO constituents through the thick peptidoglycan layer of Gram‐positive bacteria, resulting in a multi‐targeted antimicrobial response (Gu 2023; Ji et al. 2023). Although chemically distinct from GEO, SEO contains monoterpenes such as α‐thujone, camphor, and 1,8‐cineole, which destabilize membranes and interfere with energy metabolism (Císarová et al. 2018; Yazgan 2020), complementing the acid‐mediated stress induced by SUL. For Gram‐negative bacteria ( *E. coli* , *P. fluorescens* ) and salmon microbiota, the SUL and TEO combination showed the strongest synergistic effect (FICI = 0.38), resulting in a 4‐fold reduction in the MIC of SUL and an 8‐fold reduction in the MIC of TEO, compared to MICs of the antimicrobials when used alone. This enhanced AA can be attributed to the ability of SUL to partially destabilize the outer membrane of Gram‐negative bacteria, thereby facilitating the penetration and membrane‐active effects of thymol‐rich TEO. Together, these findings indicate that SUL not only contributes direct antimicrobial activity but also acts as an effective synergistic partner when combined with specific EOs, enabling substantial reductions in the required doses of both antimicrobial components while maintaining broad‐spectrum efficacy against Gram‐positive bacteria, Gram‐negative bacteria, and complex salmon microbiota.

**TABLE 5:** Fractional inhibition concentration index (FICI) values for combinations of Oregano (OEO), Thyme (TEO), Garlic (GEO), Sage (SEO), Marjoram (MEO), Laurel (LEO), Rosemary (REO) EOs and SUL.*

Bacteria	Combinations	MIC alone (mg/mL)		MIC in combination (mg/mL)		FIC		FICI	Effect
		SUL	EO	SUL	EO	SUL	EO		
*S. aureus*	SUL + OEO	3.750	0.313	0.940	0.075	0.25	0.24	0.49^c^	Synergistic
	SUL + TEO	3.750	0.313	0.940	0.075	0.25	0.25	0.50^c^	Synergistic
	SUL + GEO	3.750	0.313	0.230	0.040	0.06	0.13	0.19^a^	Synergistic
	SUL + SEO	3.750	0.313	0.230	0.040	0.06	0.13	0.19^a^	Synergistic
	SUL + MEO	3.750	0.625	0.940	0.160	0.25	0.25	0.50^c^	Synergistic
	SUL + LEO	3.750	0.625	0.940	0.160	0.25	0.25	0.50^c^	Synergistic
	SUL + REO	3.750	0.625	0.940	0.160	0.25	0.25	0.50^c^	Synergistic
*L. innocua*	SUL + OEO	3.750	0.313	0.940	0.075	0.25	0.24	0.49^c^	Synergistic
	SUL + TEO	3.750	0.313	0.940	0.075	0.25	0.25	0.50^c^	Synergistic
	SUL + GEO	3.750	0.313	0.230	0.040	0.06	0.13	0.19^a^	Synergistic
	SUL + SEO	3.750	0.313	0.230	0.040	0.06	0.13	0.19^a^	Synergistic
	SUL + MEO	3.750	0.625	0.940	0.160	0.25	0.25	0.50^c^	Synergistic
	SUL + LEO	3.750	0.625	0.940	0.160	0.25	0.25	0.50^c^	Synergistic
	SUL + REO	3.750	0.625	0.940	0.160	0.25	0.25	0.50^c^	Synergistic
*B. thermosphacta*	SUL + OEO	3.750	0.313	0.940	0.075	0.25	0.24	0.49^c^	Synergistic
	SUL + TEO	3.750	0.313	0.940	0.075	0.25	0.25	0.50^c^	Synergistic
	SUL + GEO	3.750	0.313	0.230	0.040	0.06	0.13	0.19^a^	Synergistic
	SUL + SEO	3.750	0.313	0.230	0.040	0.06	0.13	0.19^a^	Synergistic
	SUL + MEO	3.750	0.625	0.940	0.160	0.25	0.25	0.50^c^	Synergistic
	SUL + LEO	3.750	0.625	0.940	0.160	0.25	0.25	0.50^c^	Synergistic
	SUL + REO	3.750	0.625	0.940	0.160	0.25	0.25	0.50^c^	Synergistic
LAB	SUL + OEO	3.750	0.313	0.940	0.075	0.25	0.24	0.49^c^	Synergistic
	SUL + TEO	3.750	0.313	0.940	0.075	0.25	0.25	0.50^c^	Synergistic
	SUL + GEO	3.750	0.313	0.230	0.040	0.06	0.13	0.19^a^	Synergistic
	SUL + SEO	3.750	0.313	0.230	0.040	0.06	0.13	0.19^a^	Synergistic
	SUL + MEO	3.750	0.625	0.940	0.160	0.25	0.25	0.50^c^	Synergistic
	SUL + LEO	3.750	0.625	0.940	0.160	0.25	0.25	0.50^c^	Synergistic
	SUL + REO	3.750	0.625	0.940	0.160	0.25	0.25	0.50^c^	Synergistic
*E. coli*	SUL + OEO	3.750	0.313	0.940	0.075	0.25	0.24	0.49^c^	Synergistic
	SUL + TEO	3.750	0.625	0.940	0.075	0.25	0.13	0.38^b^	Synergistic
	SUL + GEO	3.750	0.625	0.940	0.160	0.25	0.25	0.50^c^	Synergistic
	SUL + SEO	3.750	0.625	0.940	0.160	0.25	0.25	0.50^c^	Synergistic
	SUL + MEO	3.750	0.625	0.940	0.160	0.25	0.25	0.50^c^	Synergistic
	SUL + LEO	3.750	0.625	0.940	0.160	0.25	0.25	0.50^c^	Synergistic
	SUL + REO	3.750	0.625	0.940	0.160	0.25	0.25	0.50^c^	Synergistic
*P. fluorescens*	SUL + OEO	3.750	0.313	0.940	0.075	0.25	0.24	0.49^c^	Synergistic
	SUL + TEO	3.750	0.625	0.940	0.075	0.25	0.13	0.38^b^	Synergistic
	SUL + GEO	3.750	0.625	0.940	0.160	0.25	0.25	0.50^c^	Synergistic
	SUL + SEO	3.750	0.625	0.940	0.160	0.25	0.25	0.50^c^	Synergistic
	SUL + MEO	3.750	0.625	0.940	0.160	0.25	0.25	0.50^c^	Synergistic
	SUL + LEO	3.750	0.625	0.940	0.160	0.25	0.25	0.50^c^	Synergistic
	SUL + REO	3.750	0.625	0.940	0.160	0.25	0.25	0.50^c^	Synergistic
Salmon microbiota	SUL + OEO	3.750	0.313	0.940	0.075	0.25	0.24	0.49^c^	Synergistic
	SUL + TEO	3.750	0.625	0.940	0.075	0.25	0.13	0.38^b^	Synergistic
	SUL + GEO	3.750	0.625	0.940	0.160	0.25	0.25	0.50^c^	Synergistic
	SUL + SEO	3.750	0.625	0.940	0.160	0.25	0.25	0.50^c^	Synergistic
	SUL + MEO	3.750	0.625	0.940	0.160	0.25	0.25	0.50^c^	Synergistic
	SUL + LEO	3.750	0.625	0.940	0.160	0.25	0.25	0.50^c^	Synergistic
	SUL + REO	3.750	0.625	0.940	0.160	0.25	0.25	0.50^c^	Synergistic

*Note:* Different superscript letters in FICI indicate statistically significant differences (*p* < 0.05), based on comparisons performed across all combinations and all bacteria tested.

* All values are means of triplicate measurements from two independent experiments (*n* = 6).

The findings from both ART+EOs and SUL + EOs combinations demonstrate that combining organic acid‐based systems with EOs markedly amplifies antimicrobial efficacy by enabling substantial MIC reductions for both components when in combination compared to MICs of the antimicrobials when used alone. The reduction on the concentration of antimicrobials for food or food‐packaging applications would be advantageous in limiting sensory impacts often associated with the use of higher EO concentrations to improve microbial safety, reducing formulation costs and complying with regulatory limits, while maintaining or enhancing antimicrobial performance (Angane et al. 2024; Kamau et al. 2025; dos Santos et al. 2025). The consistent synergistic effect observed in this study across diverse bacterial groups further underscores the potential of weak organic acid–EO combinations as versatile, broad‐spectrum antimicrobial strategies suitable for modern food preservation systems.

##### 3.3.2.3 EO‐EO Combinations

Combinations of EOs (OEO, TEO, GEO, SEO, MEO, LEO or REO) resulted in 19 different pairwise EO‐EO combinations, with all combinations having a synergistic (FICI ≤ 0.5) effect and significantly (*p* < 0.05) reducing the antimicrobial concentrations of each EO combined (Table 6). Among the tested combinations, the TEO and OEO combination exhibited the strongest synergistic (FICI = 0.36) effect against *E. coli* , *P. fluorescens* and salmon microbiota, resulting in an 8‐fold reduction in the MIC of TEO and a 4‐fold reduction in the MIC of OEO compared to MICs of the antimicrobials when used alone. This synergistic effect may be attributed to the complementary actions of thymol and carvacrol, the dominant phenolic constituents of TEO and OEO, respectively. Both compounds integrate into lipid bilayers and disrupt membrane fluidity; however, subtle structural differences enable cooperative destabilization of the bacterial membrane, leading to intensified leakage of ions and intracellular contents when applied in combination (Magi et al. 2015; Moghrovyan et al. 2024; Radocchia et al. 2025; Tao et al. 2025). Comparable thymol–carvacrol synergistic effect, with FICI values well below the synergistic effect threshold (≤ 0.5), has been reported against both Gram‐positive and Gram‐negative bacteria, supporting the mechanistic basis of the present results (Ermenlieva 2025; Radocchia et al. 2025; Tao et al. 2025). The consistent synergistic activity observed across individual strains and the complex salmon microbiota further suggests that EO combinations exert broad‐spectrum antimicrobial effects that may be less susceptible to bacterial adaptation (Dosoky and Setzer 2018; Heghes et al. 2019). In agreement with the present findings, synergistic effects has been demonstrated for several EO combinations against Gram‐positive and Gram‐negative bacteria, including OEO + REO (de Azeredo et al. 2011) and SEO + TEO (Mokhtari et al. 2023), as well as other EO mixtures with FICI values ≤ 0.5 (Angane et al. 2024; El‐Zehery et al. 2022; Kim et al. 2021; Purkait et al. 2018). Synergistic interactions among EOs are largely attributed to the presence of diverse bioactive compounds with distinct chemical structures, allowing whole‐oil mixtures to exert effects that exceed those of individual constituents (Kamau et al. 2025). For instance, the concurrent action of phenolic monoterpenes, flavonoids, sulfur compounds and other minor constituents can collectively compromise bacterial membrane integrity, increase cytoplasmic permeability and interfere with key stress response systems, including efflux activity and oxidative defense pathways, potentiating overall AA (Angane et al. 2024; Kim et al. 2021). Such combined effects ultimately reduce the concentrations required to inhibit bacterial growth (Haleem et al. 2019). In addition to biochemical interactions at the membrane level, synergistic effect is further influenced by physicochemical parameters including solubility, lipophilicity and functional group interactions among EO constituents (Peng et al. 2019a, 2019b). The ultrasonication‐based emulsification approach employed in this study likely contributed to these effects by reducing particle size and improving EO dispersion and uniformity, thereby increasing bioavailability and antimicrobial efficacy while potentially limiting the development of antimicrobial resistance (Ambrosio et al. 2017; Kamau et al. 2025). Collectively, these findings support growing evidence that rational EO combination represents an effective hurdle strategy, enabling strong AA at reduced EO concentrations. This approach is particularly relevant for food systems, where lowering total EO load is critical for preserving sensory quality and meeting regulatory requirements while ensuring microbial safety (Angane et al. 2024; Kaur et al. 2024; Mazumder et al. 2026; Stamova et al. 2025).

**TABLE 6:** Fractional inhibition concentration index (FICI) values of EO‐EO combinations between Oregano (OEO), Thyme (TEO), Garlic (GEO), Sage (SEO), Marjoram (MEO), Laurel (LEO) or Rosemary (REO) EOs.*

Bacteria	Combinations	MIC alone (mg/mL)		MIC in combination (mg/mL)		FIC		FICI	Effect
	EO_1_ + EO_2_	EO_1_	EO_2_	EO_1_	EO_2_	EO_1_	EO_2_		
*S. aureus*	TEO + OEO	0.313	0.313	0.075	0.075	0.24	0.24	0.48^c^	Synergistic
	TEO + GEO	0.313	0.313	0.075	0.075	0.24	0.24	0.48^c^	Synergistic
	TEO + SEO	0.313	0.313	0.075	0.075	0.24	0.24	0.48^c^	Synergistic
	OEO + GEO	0.313	0.313	0.075	0.075	0.24	0.24	0.48^c^	Synergistic
	OEO + SEO	0.313	0.313	0.075	0.075	0.24	0.24	0.48^c^	Synergistic
	OEO + MEO	0.313	0.625	0.075	0.160	0.24	0.26	0.50^d^	Synergistic
	OEO + LEO	0.313	0.625	0.075	0.160	0.24	0.26	0.50^d^	Synergistic
	OEO + REO	0.313	0.625	0.075	0.160	0.24	0.26	0.50^d^	Synergistic
	TEO + MEO	0.313	0.625	0.075	0.075	0.26	0.13	0.38^b^	Synergistic
	TEO + LEO	0.313	0.625	0.075	0.075	0.26	0.13	0.38^b^	Synergistic
	TEO + REO	0.313	0.625	0.075	0.075	0.26	0.13	0.38^b^	Synergistic
	GEO + MEO	0.313	0.625	0.075	0.075	0.26	0.13	0.38^b^	Synergistic
	GEO + LEO	0.313	0.625	0.075	0.075	0.26	0.13	0.38^b^	Synergistic
	GEO + REO	0.313	0.625	0.075	0.075	0.26	0.13	0.38^b^	Synergistic
	SEO + MEO	0.313	0.625	0.075	0.075	0.26	0.13	0.38^b^	Synergistic
	SEO + LEO	0.313	0.625	0.075	0.075	0.26	0.13	0.38^b^	Synergistic
	SEO + REO	0.313	0.625	0.075	0.075	0.26	0.13	0.38^b^	Synergistic
	MEO + LEO	0.625	0.625	0.160	0.160	0.26	0.26	0.51^d^	Synergistic
	MEO + REO	0.625	0.625	0.160	0.160	0.26	0.26	0.51^d^	Synergistic
*L. innocua*	TEO + OEO	0.313	0.313	0.075	0.075	0.24	0.24	0.48^c^	Synergistic
	TEO + GEO	0.313	0.313	0.075	0.075	0.24	0.24	0.48^c^	Synergistic
	TEO + SEO	0.313	0.313	0.075	0.075	0.24	0.24	0.48^c^	Synergistic
	OEO + GEO	0.313	0.313	0.075	0.075	0.24	0.24	0.48^c^	Synergistic
	OEO + SEO	0.313	0.313	0.075	0.075	0.24	0.24	0.48^c^	Synergistic
	OEO + MEO	0.313	0.625	0.075	0.160	0.24	0.26	0.50^d^	Synergistic
	OEO + LEO	0.313	0.625	0.075	0.160	0.24	0.26	0.50^d^	Synergistic
	OEO + REO	0.313	0.625	0.075	0.160	0.24	0.26	0.50^d^	Synergistic
	TEO + MEO	0.313	0.625	0.075	0.075	0.26	0.13	0.38^b^	Synergistic
	TEO + LEO	0.313	0.625	0.075	0.075	0.26	0.13	0.38^b^	Synergistic
	TEO + REO	0.313	0.625	0.075	0.075	0.26	0.13	0.38^b^	Synergistic
	GEO + MEO	0.313	0.625	0.075	0.075	0.26	0.13	0.38^b^	Synergistic
	GEO + LEO	0.313	0.625	0.075	0.075	0.26	0.13	0.38^b^	Synergistic
	GEO + REO	0.313	0.625	0.075	0.075	0.26	0.13	0.38^b^	Synergistic
	SEO + MEO	0.313	0.625	0.075	0.075	0.26	0.13	0.38^b^	Synergistic
	SEO + LEO	0.313	0.625	0.075	0.075	0.26	0.13	0.38^b^	Synergistic
	SEO + REO	0.313	0.625	0.075	0.075	0.26	0.13	0.38^b^	Synergistic
	MEO + LEO	0.625	0.625	0.160	0.160	0.26	0.26	0.51^d^	Synergistic
	MEO + REO	0.625	0.625	0.160	0.160	0.26	0.26	0.51^d^	Synergistic
*B. thermosphacta*	TEO + OEO	0.313	0.313	0.075	0.075	0.24	0.24	0.48^c^	Synergistic
	TEO + GEO	0.313	0.313	0.075	0.075	0.24	0.24	0.48^c^	Synergistic
	TEO + SEO	0.313	0.313	0.075	0.075	0.24	0.24	0.48^c^	Synergistic
	OEO + GEO	0.313	0.313	0.075	0.075	0.24	0.24	0.48^c^	Synergistic
	OEO + SEO	0.313	0.313	0.075	0.075	0.24	0.24	0.48^c^	Synergistic
	OEO + MEO	0.313	0.625	0.075	0.160	0.24	0.26	0.50^d^	Synergistic
	OEO + LEO	0.313	0.625	0.075	0.160	0.24	0.26	0.50^d^	Synergistic
	OEO + REO	0.313	0.625	0.075	0.160	0.24	0.26	0.50^d^	Synergistic
	TEO + MEO	0.313	0.625	0.075	0.075	0.26	0.13	0.38^b^	Synergistic
	TEO + LEO	0.313	0.625	0.075	0.075	0.26	0.13	0.38^b^	Synergistic
	TEO + REO	0.313	0.625	0.075	0.075	0.26	0.13	0.38^b^	Synergistic
	GEO + MEO	0.313	0.625	0.075	0.075	0.26	0.13	0.38^b^	Synergistic
	GEO + LEO	0.313	0.625	0.075	0.075	0.26	0.13	0.38^b^	Synergistic
	GEO + REO	0.313	0.625	0.075	0.075	0.26	0.13	0.38^b^	Synergistic
	SEO + MEO	0.313	0.625	0.075	0.075	0.26	0.13	0.38^b^	Synergistic
	SEO + LEO	0.313	0.625	0.075	0.075	0.26	0.13	0.38^b^	Synergistic
	SEO + REO	0.313	0.625	0.075	0.075	0.26	0.13	0.38^b^	Synergistic
	MEO + LEO	0.625	0.625	0.160	0.160	0.26	0.26	0.51^d^	Synergistic
	MEO + REO	0.625	0.625	0.160	0.160	0.26	0.26	0.51^d^	Synergistic
LAB	TEO + OEO	0.313	0.313	0.075	0.075	0.24	0.24	0.48^c^	Synergistic
	TEO + GEO	0.313	0.313	0.075	0.075	0.24	0.24	0.48^c^	Synergistic
	TEO + SEO	0.313	0.313	0.075	0.075	0.24	0.24	0.48^c^	Synergistic
	OEO + GEO	0.313	0.313	0.075	0.075	0.24	0.24	0.48^c^	Synergistic
	OEO + SEO	0.313	0.313	0.075	0.075	0.24	0.24	0.48^c^	Synergistic
	OEO + MEO	0.313	0.625	0.075	0.160	0.24	0.26	0.50^d^	Synergistic
	OEO + LEO	0.313	0.625	0.075	0.160	0.24	0.26	0.50^d^	Synergistic
	OEO + REO	0.313	0.625	0.075	0.160	0.24	0.26	0.50^d^	Synergistic
	TEO + MEO	0.313	0.625	0.075	0.075	0.26	0.13	0.38^b^	Synergistic
	TEO + LEO	0.313	0.625	0.075	0.075	0.26	0.13	0.38^b^	Synergistic
	TEO + REO	0.313	0.625	0.075	0.075	0.26	0.13	0.38^b^	Synergistic
	GEO + MEO	0.313	0.625	0.075	0.075	0.26	0.13	0.38^b^	Synergistic
	GEO + LEO	0.313	0.625	0.075	0.075	0.26	0.13	0.38^b^	Synergistic
	GEO + REO	0.313	0.625	0.075	0.075	0.26	0.13	0.38^b^	Synergistic
	SEO + MEO	0.313	0.625	0.075	0.075	0.26	0.13	0.38^b^	Synergistic
	SEO + LEO	0.313	0.625	0.075	0.075	0.26	0.13	0.38^b^	Synergistic
	SEO + REO	0.313	0.625	0.075	0.075	0.26	0.13	0.38^b^	Synergistic
	MEO + LEO	0.625	0.625	0.160	0.160	0.26	0.26	0.51^d^	Synergistic
	MEO + REO	0.625	0.625	0.160	0.160	0.26	0.26	0.51^d^	Synergistic
*E. coli*	TEO + OEO	0.630	0.313	0.075	0.075	0.12	0.24	0.36^a^	Synergistic
	TEO + GEO	0.625	0.625	0.075	0.160	0.13	0.26	0.38^b^	Synergistic
	TEO + SEO	0.625	0.625	0.075	0.160	0.13	0.26	0.38^b^	Synergistic
	OEO + GEO	0.313	0.625	0.075	0.075	0.26	0.13	0.38^b^	Synergistic
	OEO + SEO	0.313	0.625	0.075	0.075	0.26	0.13	0.38^b^	Synergistic
	OEO + MEO	0.313	0.625	0.075	0.160	0.26	0.26	0.51^d^	Synergistic
	OEO + LEO	0.313	0.625	0.075	0.160	0.26	0.26	0.51^d^	Synergistic
	OEO + REO	0.313	0.625	0.075	0.160	0.26	0.26	0.51^d^	Synergistic
	TEO + MEO	0.630	0.625	0.160	0.160	0.25	0.26	0.51^d^	Synergistic
	TEO + LEO	0.630	0.625	0.160	0.160	0.25	0.26	0.51^d^	Synergistic
	TEO + REO	0.630	0.625	0.160	0.160	0.25	0.26	0.51^d^	Synergistic
	GEO + MEO	0.630	0.625	0.160	0.160	0.25	0.26	0.51^d^	Synergistic
	GEO + LEO	0.630	0.625	0.160	0.160	0.25	0.26	0.51^d^	Synergistic
	GEO + REO	0.630	0.625	0.160	0.160	0.25	0.26	0.51^d^	Synergistic
	SEO + MEO	0.630	0.625	0.160	0.160	0.25	0.26	0.51^d^	Synergistic
	SEO + LEO	0.630	0.625	0.160	0.160	0.25	0.26	0.51^d^	Synergistic
	SEO + REO	0.630	0.625	0.160	0.160	0.25	0.26	0.51^d^	Synergistic
	MEO + LEO	0.625	0.625	0.160	0.160	0.26	0.26	0.51^d^	Synergistic
	MEO + REO	0.625	0.625	0.160	0.160	0.26	0.26	0.51^d^	Synergistic
*P. fluorescens*	TEO + OEO	0.630	0.313	0.075	0.075	0.12	0.24	0.36^a^	Synergistic
	TEO + GEO	0.625	0.625	0.075	0.160	0.13	0.26	0.38^b^	Synergistic
	TEO + SEO	0.625	0.625	0.075	0.160	0.13	0.26	0.38^b^	Synergistic
	OEO + GEO	0.313	0.625	0.075	0.075	0.26	0.13	0.38^b^	Synergistic
	OEO + SEO	0.313	0.625	0.075	0.075	0.26	0.13	0.38^b^	Synergistic
	OEO + MEO	0.313	0.625	0.075	0.160	0.26	0.26	0.51^d^	Synergistic
	OEO + LEO	0.313	0.625	0.075	0.160	0.26	0.26	0.51^d^	Synergistic
	OEO + REO	0.313	0.625	0.075	0.160	0.26	0.26	0.51^d^	Synergistic
	TEO + MEO	0.630	0.625	0.160	0.160	0.25	0.26	0.51^d^	Synergistic
	TEO + LEO	0.630	0.625	0.160	0.160	0.25	0.26	0.51^d^	Synergistic
	TEO + REO	0.630	0.625	0.160	0.160	0.25	0.26	0.51^d^	Synergistic
	GEO + MEO	0.630	0.625	0.160	0.160	0.25	0.26	0.51^d^	Synergistic
	GEO + LEO	0.630	0.625	0.160	0.160	0.25	0.26	0.51^d^	Synergistic
	GEO + REO	0.630	0.625	0.160	0.160	0.25	0.26	0.51^d^	Synergistic
	SEO + MEO	0.630	0.625	0.160	0.160	0.25	0.26	0.51^d^	Synergistic
	SEO + LEO	0.630	0.625	0.160	0.160	0.25	0.26	0.51^d^	Synergistic
	SEO + REO	0.630	0.625	0.160	0.160	0.25	0.26	0.51^d^	Synergistic
	MEO + LEO	0.625	0.625	0.160	0.160	0.26	0.26	0.51^d^	Synergistic
	MEO + REO	0.625	0.625	0.160	0.160	0.26	0.26	0.51^d^	Synergistic
Salmon microbiota	TEO + OEO	0.630	0.313	0.075	0.075	0.12	0.24	0.36^a^	Synergistic
	TEO + GEO	0.625	0.625	0.075	0.160	0.13	0.26	0.38^b^	Synergistic
	TEO + SEO	0.625	0.625	0.075	0.160	0.13	0.26	0.38^b^	Synergistic
	OEO + GEO	0.313	0.625	0.075	0.075	0.26	0.13	0.38^b^	Synergistic
	OEO + SEO	0.313	0.625	0.075	0.075	0.26	0.13	0.38^b^	Synergistic
	OEO + MEO	0.313	0.625	0.075	0.160	0.26	0.26	0.51^d^	Synergistic
	OEO + LEO	0.313	0.625	0.075	0.160	0.26	0.26	0.51^d^	Synergistic
	OEO + REO	0.313	0.625	0.075	0.160	0.26	0.26	0.51^d^	Synergistic
	TEO + MEO	0.630	0.625	0.160	0.160	0.25	0.26	0.51^d^	Synergistic
	TEO + LEO	0.630	0.625	0.160	0.160	0.25	0.26	0.51^d^	Synergistic
	TEO + REO	0.630	0.625	0.160	0.160	0.25	0.26	0.51^d^	Synergistic
	GEO + MEO	0.630	0.625	0.160	0.160	0.25	0.26	0.51^d^	Synergistic
	GEO + LEO	0.630	0.625	0.160	0.160	0.25	0.26	0.51^d^	Synergistic
	GEO + REO	0.630	0.625	0.160	0.160	0.25	0.26	0.51^d^	Synergistic
	SEO + MEO	0.630	0.625	0.160	0.160	0.25	0.26	0.51^d^	Synergistic
	SEO + LEO	0.630	0.625	0.160	0.160	0.25	0.26	0.51^d^	Synergistic
	SEO + REO	0.630	0.625	0.160	0.160	0.25	0.26	0.51^d^	Synergistic
	MEO + LEO	0.625	0.625	0.160	0.160	0.26	0.26	0.51^d^	Synergistic
	MEO + REO	0.625	0.625	0.160	0.160	0.26	0.26	0.51^d^	Synergy

*Note:* Different superscripts letters in FICI indicate statistically significant differences (*p* < 0.05), based on comparisons performed across all combinations and all bacteria tested.

* All values are means of triplicate measurements from two independent experiments (*n* = 6).

#### 3.3.3 Time‐Kill Kinetics Test

**FIGURE 3:**
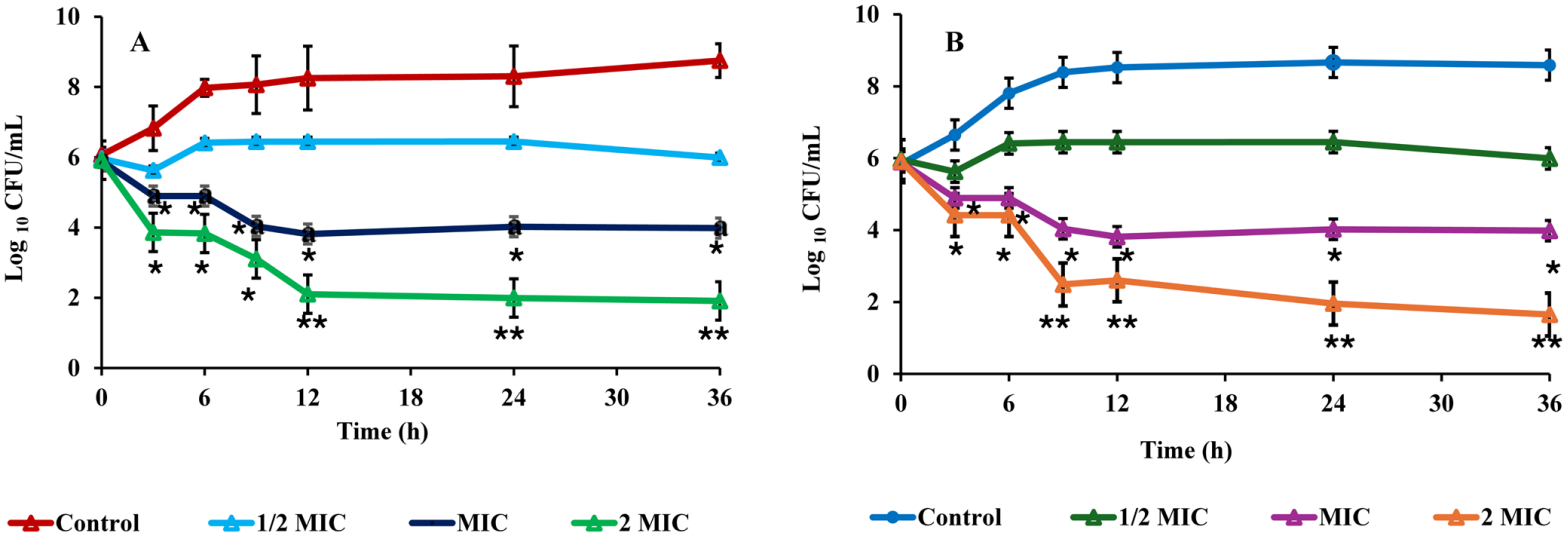
Time‐kill kinetics of Oregano/Thyme EO against (A) *P. fluorescens* and (B) *S. aureus* . Concentrations included 0 (control), ½ MIC (0.04 mg/mL OEO + 0.04 mg/mL TEO), MIC (0.08 mg/mL OEO + 0.08 mg/mL TEO), and 2×MIC (0.16 mg/mL OEO + 0.16 mg/mL TEO). *p < 0.05 and **p < 0.01 indicate statistically significant decreases in bacterial populations at the corresponding sampling hours compared with the baseline count measured on day 0.

**FIGURE 4:**
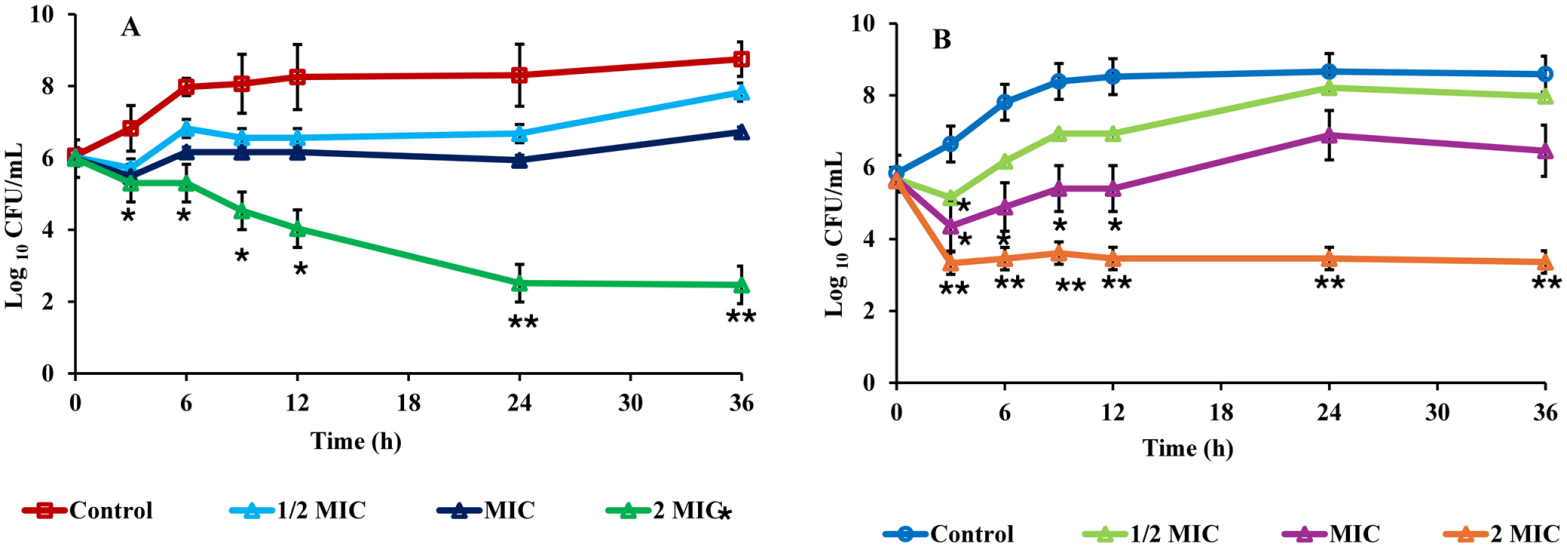
Time‐kill kinetics of Chitosan/Garlic EO against (A) *P. fluorescens* and (B) *S. aureus* . Concentrations included 0 (control), ½ MIC (0.08 mg/mL TEO + 0.02 mg/mL CS), MIC (0.16 mg/mL TEO + 0.03 mg/mL CS) and 2×MIC (0.31 mg/mL TEO + 0.06 mg/mL CS). *p < 0.05 and **p < 0.01 indicate statistically significant decreases in bacterial populations at the corresponding sampling hours compared with the baseline count measured on day 0.

Time–kill kinetics assays provide dynamic insight into the antimicrobial action of test agents over time, enabling differentiation between bacteriostatic and bactericidal effects (CLSI 1999). Unlike endpoint MIC determinations, this approach captures temporal changes in bacterial viability and therefore, offers a more comprehensive assessment of antimicrobial efficacy (Israyilova et al. 2022). To validate the antimicrobial performance and killing kinetics of NAM combinations that showed synergistic effects (OEO + TEO, TEO + SUL, TEO + ART, and CS + GEO), a standardized in vitro time–kill assay against *S. aureus* and *P. fluorescens* was carried out. Independent of the tested bacteria, the antimicrobial combination OEO + TEO exhibited bacteriostatic activity at concentrations 0.5×MIC_comb_ and MIC_comb_, whereas a bactericidal effect was achieved at 2×MIC_comb_, as evidenced by a 99.9% (≥ 3 Log_10_ reduction in viable cell counts within 24 h post‐inoculation; Figure 3). The pronounced decline in bacterial viable populations at higher concentrations (> 1×MIC) indicates that cell viability was concentration‐dependent, where increased EO levels accelerated bacterial cell death and reinforced a dose–response relationship. Comparable bactericidal effect against foodborne and spoilage bacteria have been reported for other EO combinations with synergistic effect, including oregano–rosemary (de Azeredo et al. 2011), clove–rosemary (Fu et al. 2007), *Zataria multiflora*– *Origanum vulgare* (Kakhki et al. 2020), oregano–marjoram–coriander (Kraśniewska et al. 2020), and peppermint–thyme (Angane et al. 2024). The bactericidal activity of EO combinations can be attributed to their volatile bioactive constituents, which act synergistically to disrupt membrane integrity, increase permeability, and ultimately induce cell death. Such mechanisms are particularly relevant for intrinsically resilient Gram‐negative bacteria such as *P. fluorescens* . The presence of a double‐layered phospholipid membrane, characteristic of Gram‐negative bacteria, may confer increased resistance to NAM agents (El‐Zehery et al. 2022; Yousefi et al. 2020). Similar results were observed for the antimicrobial combination CS + GEO, which also exhibited a clear concentration‐dependent inhibitory effect. *P. fluorescens* bacterial cell decreased significantly (*p* < 0.05) over time at when the concentration of this antimicrobial combination was 2×MICcomb, showing an evident bactericidal effect 24 h post‐inoculation (Figure 4A). This pronounced bactericidal effect may be attributed to a complimentary antimicrobial activity mechanism of the antimicrobials used as CS has been reported to have the ability to interfere with membrane‐associated transport systems, particularly through inhibition of bacterial efflux pumps, a critical resistance mechanism in Gram‐negative bacteria such as *P. fluorescens* (Oliveira et al. 2024). In addition, the incorporation of EOs into positively charged CS matrices can generate a semi‐permeable barrier that restricts nutrient exchange, thereby reducing cellular respiration and delaying bacterial growth (Raphaël and Meimandipour 2017). In contrast, no bactericidal effect was observed against *S. aureus* , with reductions remaining below 3 log_10_ CFU/mL (Figure 4B). This reduced susceptibility may be linked to the presence of small‐colony variants, which exhibit decreased metabolic activity and a diminished electrochemical gradient, rendering them inherently more tolerant to chitosan than parental strains (Raafat et al. 2008). Nevertheless, the absence of bacterial regrowth after 36 h indicates sustained antimicrobial pressure, suggesting that the EO–chitosan system not only enhances antimicrobial efficacy but may also limit the emergence of resistance, in agreement with previous reports (El‐Zehery et al. 2022; Oliveira et al. 2024).

**FIGURE 5:**
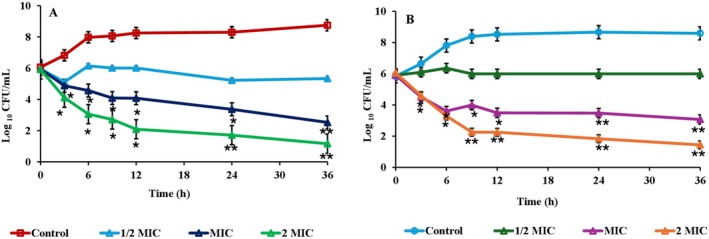
Time‐kill kinetics of Sulac/Thyme EO against (A) *P. fluorescens* and (B) *S. aureus* . Concentrations included 0 (control), ½ MIC (0.47 mg/mL SUL + 0.04 mg/mL TEO), MIC (0.94 mg/mL SUL + 0.08 mg/mL TEO), and 2×MIC (1.88 mg/mL SUL + 0.16 mg/mL TEO).*p < 0.05 and **p < 0.01 indicate statistically significant decreases in bacterial populations at the corresponding sampling hours compared with the baseline count measured on day 0.

**FIGURE 6:**
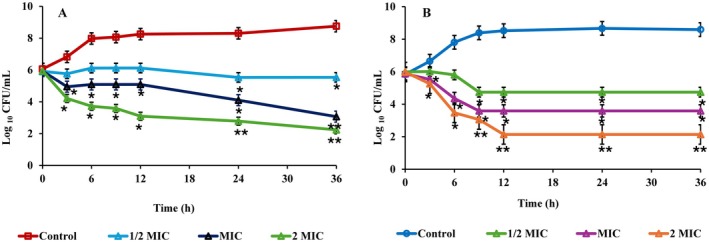
Time‐kill kinetics of Articoat/Thyme EO against (A) *P. fluorescens* and (B) *S. aureus* . Concentrations included 0 (control), ½ MIC (3.13 mg/mL ART + 0.03 mg/mL TEO), MIC (6.25 mg/mL ART + 0.07 mg/mL TEO) and 2×MIC (12.5 mg/mL ART + 0.14 mg/mL TEO). *p < 0.05 and **p < 0.01 indicate statistically significant decreases in bacterial populations at the corresponding sampling hours compared with the baseline count measured on day 0.

The combination of antimicrobials TEO + SUL demonstrated a rapid and sustained bactericidal effect, achieving a ≥ 3 Log_10_ reduction (99.9% killing) in viable counts of both *S. aureus* and *P. fluorescens* within 12 h of exposure at a concentration 2×MICcomb (Figure 5). In contrast, the TEO + ART combination at the same concentration produced a comparable bactericidal effect only after prolonged exposure, with complete killing observed at 36 h (Figure 6). These findings are consistent with earlier studies reporting enhanced antimicrobial efficacy when EOs are combined with weak organic acids (de Souza et al. 2009). The observed bactericidal activity can be attributed to the synergistic interplay between weak organic acids, which disrupt intracellular pH homeostasis, and EOs, which compromise membrane integrity and permeability. Notably, this study is the first to demonstrate the inhibitory effects of simultaneous application of EO with SUL or ART at sub‐inhibitory concentrations against *S. aureus* and *P. fluorescens* , as confirmed by both FIC index determination and time‐kill kinetics. Accordingly, the combined use of EOs and weak organic acids including commercially available mixtures of weak acids (SUL or ART) represents a promising natural preservation strategy for food systems, offering an effective additional hurdle to limit the survival and proliferation of pathogenic and spoilage microorganisms.

## 4 Conclusions

This study demonstrated that the NAM combinations (ART–EOs, CS–EOs, SUL–EOs, and EO–EO) can significantly (*p* < 0.05) reduce antimicrobial concentrations used against pure strains of Gram‐positive ( *S. aureus* , *L. innocua* , *B. thermosphacta* , and LAB), Gram‐negative ( *E. coli* , *P. fluorescens* ) bacteria, and salmon microbiota when compared to the MIC concentrations when each antimicrobial is used alone. The best NAM combination against Gram‐positive bacteria was CS combined with GEO, which reduced (*p* < 0.05) MICs of GEO and CS by 16 and 8 fold, respectively. While the synergistic combination of SUL with GEO or SEO significantly (*p* < 0.05) reduced the MIC concentration of SUL by 17 fold and those of the corresponding EOs by 8 fold to have a similar effect when compared to their MIC when each antimicrobial is used alone. Against Gram‐negative bacteria and salmon microbiota, the synergistic effect of the combinations ART‐OEO, ART‐TEO, and OEO‐TEO had a strong synergistic effect, which decreased (*p* < 0.05) MICs of each antimicrobial in combination by up to 8 fold when compared to their MIC when each antimicrobial is used alone. The time‐kill assay confirmed the bactericidal effect of the NAM combinations (ART–EOs, SUL–EOs, and EO–EO) against *S. aureus* and *P. fluorescens* . Overall, the synergistic effect of the NAM combinations studied exhibited broad spectrum antimicrobial activity not only against pure culture Gram‐positive and Gram‐negative bacteria but also against salmon microbiota.

Results of this study highlight that different antimicrobial mechanisms of NAM tested could be exploited to enhance antimicrobial efficacy through synergistic interactions and these systems can be integrated with other antimicrobial agents. Such multi‐mechanistic combinations represent promising clean label alternatives to synthetic preservatives, enabling the use of lower antimicrobial loads while minimizing potential toxicity concerns and reducing the risk of bacterial resistance. The observed reductions in required antimicrobial concentrations are particularly relevant for food and food packaging applications, where they may help mitigate sensory impacts, support regulatory compliance, and facilitate integration into clean label preservation strategies. More in vivo studies are needed to validate the performance of these combinations within real food matrices and to assess their suitability as part of a broader hurdle technology approach aimed at improving microbial safety, extending shelf‐life, and maintaining product quality.

## Author Contributions

**Michael A. Morris:** formal analysis, project administration, supervision, conceptualization. **Joe P. Kerry:** supervision, project administration, writing – review and editing, data curation, resources, funding acquisition, investigation, conceptualization. **Paul G. Kamau:** writing – original draft, investigation, conceptualization, methodology, data curation, visualization, validation. **Paola C. Alzate:** methodology, validation, visualization, supervision, data curation, investigation, conceptualization. **Malco C. Cruz‐Romero:** writing – review and editing, validation, visualization, methodology, formal analysis, investigation, conceptualization, supervision, data curation, project administration.

## Funding

This work was funded by the Food Institutional Research Measure (FIRM), grant number 2021R412, administered by the Department of Agriculture, Food and the Marine, Ireland.

## Conflicts of Interest

The authors declare no conflicts of interest.

## Supporting information


**Table S1:** Manufacturer‐reported chemical composition of essential oils used in this study. Composition data were obtained from supplier product specification sheets and are reported as relative percentage ranges (% w/w). Lionel Hitchen Ltd. (2024), Product specifications and certificates of analysis for culinary essential oils used in experimental work, Barton Stacey, Hampshire, UK.
**Table S2:** Summary of best BLAST hits per Lactic acid bacteria (LAB) as isolated from raw salmon microbiota.
**Table S3:** Summary of best BLAST hits per *B. thermosphacta* as isolated from raw salmon microbiota.

## Data Availability

The authors confirm that the data supporting the findings of this study are available within the article and/or its Supporting Information.
