# Supplementary material for: Synergistic Activity of Natural Antimicrobials Against Common Fish Spoilage and Pathogenic Bacteria
Source: Food Sci Nutr. 2026 Jul 17;14(7):e72025. doi: 10.1002/fsn3.72025 (PMC13377800; doi:10.1002/fsn3.72025)
Supplement: Supplementary file 1 — Table S1: Manufacturer‐reported chemical composition of essential oils used in this study. Composition data were obtained from supplier product specification sheets and are reported as relative percentage ranges (% w/w). Lionel Hitchen Ltd. (2024), Product specifications and certificates of analysis for culinary essential oils used in experimental work, Barton Stacey, Hampshire, UK. Table S2: Summary of best BLAST hits per Lactic acid bacteria (LAB) as isolated from raw salmon microbiota. Table S3: Summary of best BLAST hits per B. thermosphacta as isolated from raw salmon microbiota. [file FSN3-14-e72025-s001.docx]

**SUPPLEMENTARY MATERIAL**

**Table S1**. Manufacturer-reported chemical composition of essential oils used in this study. Composition data were obtained from supplier product specification sheets and are reported as relative percentage ranges (% w/w). Lionel Hitchen Ltd (2024), Product specifications and certificates of analysis for culinary essential oils used in experimental work, Barton Stacey, Hampshire, UK.

| **Essential oil (botanical source)** | **Major reported constituents (% w/w, range)** |
| --- | --- |
| Laurel leaf (*Laurus nobilis L.)* | 1,8-Cineole (50–100); α-Terpinyl acetate (10–<20); Linalool (5–<10); α-Pinene (5–<10); β-Pinene (1–<5); α-Terpineol (1–<5); Methyl eugenol (2.2); Estragole (0.1) |
| Marjoram (*Origanum majorana L*.) | Terpinen-4-ol (20–<50); Linalool (10–<20); γ-Terpinene (10–<20); d-Limonene (5–<10); α-Terpineol (5–<10); Terpinolene (1–<5); Myrcene (1–<5); β-Caryophyllene (1–<5); p-Cymene (1–<5) |
| Oregano (*Origanum vulgare L*.) | Carvacrol (50–100); p-Cymene (10–<20); γ-Terpinene (10–<20); Thymol (1–<5); Linalool (1–<5) |
| Rosemary (*Rosmarinus officinalis L*.) | α-Pinene (20–<50); Camphor (20–<50); 1,8-Cineole (10–<20); Camphene (10–<20); β-Pinene (5–<10); d-Limonene (1–<5); Myrcene (1–<5); p-Cymene (1–<5); Linalool (1–<5) |
| Sage (*Salvia officinalis L*.) | Camphor (20–<50); Camphene (5–<10); α-Pinene (1–<5); d-Limonene (1–<5); Thujone (0.33) |
| Thyme (*Thymus vulgaris L*.) | Thymol (50–100); p-Cymene (10–<20); γ-Terpinene (5–<10); Linalool (1–<5); Carvacrol (1–<5); Myrcene (1–<5); β-Caryophyllene (1–<5); α-Pinene (1–<5); Terpinen-4-ol (1–<5) |
| Garlic (*Allium sativum L*.) | Diallyl trisulfide (20–<50); Diallyl disulfide (20–<50); Dimethyl disulfide (1–<5) |

**Table S 2** Summary of best BLAST hits per Lactic acid bacteria (LAB) as isolated from raw salmon microbiota. *

| **Sample** | **Accession** | **Scientific name** | **E-value** | **Percent identity (%)** | **Query coverage (%)** |
| --- | --- | --- | --- | --- | --- |
| LAB isolate 1 | KF673498.1 | *Lacticaseibacillus casei* | 0.0 | 99.75 | 97 |
| LAB isolate 2 | PX512392.1 | *Lacticaseibacillus paracasei* | 0.0 | 99.81 | 100 |
| LAB isolate 3 | MT597689.1 | *Lacticaseibacillus paracasei* | 0.0 | 99.47 | 99 |

*Sequence similarity searches were performed using NCBI BLAST+ v2.17.0 (released July 2025) via the NCBI BLAST web interface (https://blast.ncbi.nlm.nih.gov). Ranking prioritized lowest E-value, highest Max score, Query coverage and Percent identity.

**Table S 3** Summary of best BLAST hits per B. thermosphacta as isolated from raw salmon microbiota. *

| **Sample** | **Accession** | **Scientific name** | **E-value** | **Percent identity (%)** | **Query coverage (%)** |
| --- | --- | --- | --- | --- | --- |
| *B. thermosphacta* isolate 1 | MW047723.1 | *Brochothrix sp.* | 0.0 | 99.59 | 96 |
| *B. thermosphacta* isolate 2 | LT993737.1 | *Brochothrix thermosphacta* | 0.0 | 99.84 | 95 |
| *B. thermosphacta* isolate 3 | OP164731.1 | *Brochothrix thermosphacta* | 0.0 | 99.2 | 98 |

*Sequence similarity searches were performed using NCBI BLAST+ v2.17.0 (released July 2025) via the NCBI BLAST web interface (https://blast.ncbi.nlm.nih.gov). Ranking prioritized lowest E-value, highest Max score, Query coverage and Percent identity.
